# DRB1 and DRB2 Are Required for an Appropriate miRNA-Mediated Molecular Response to Salt Stress in *Arabidopsis thaliana*

**DOI:** 10.3390/plants14060924

**Published:** 2025-03-15

**Authors:** Joseph L. Pegler, Jackson M. J. Oultram, Christopher P. L. Grof, Andrew L. Eamens

**Affiliations:** 1 Centre for Plant Science, School of Environmental and Life Sciences, College of Engineering, Science and Environment, University of Newcastle, Callaghan, NSW 2308, Australia; joseph.pegler@newcastle.edu.au (J.L.P.); jackson.oultram@newcastle.edu.au (J.M.J.O.); chris.grof@newcastle.edu.au (C.P.L.G.); 2 School of Agriculture and Food Sustainability, The University of Queensland, St Lucia, QLD 4072, Australia; 3 School of Health, University of the Sunshine Coast, Maroochydore, QLD 4558, Australia

**Keywords:** *Arabidopsis thaliana* (*Arabidopsis*), salt stress, microRNA (miRNA), gene expression regulation, DOUBLE-STRANDED RNA BINDING (DRB) protein, DRB1, DRB2

## Abstract

In plants, microRNAs (miRNAs) and their target genes have been demonstrated to form an essential component of the molecular response to salt stress. In *Arabidopsis thaliana* (*Arabidopsis*), DOUBLE-STRANDED RNA BINDING1 (DRB1) and DRB2 are required to produce specific miRNA populations throughout normal development and in response to abiotic stress. The phenotypic and physiological assessment of 15-day-old wild-type *Arabidopsis* seedlings, and of the *drb1* and *drb2* mutants following a 7-day period of salt stress, revealed the *drb2* mutant to be more sensitive to salt stress than the *drb1* mutant. However, the assessment of miRNA abundance and miRNA target gene expression showed that the ability of both *drb* mutants to mount an appropriate miRNA-mediated molecular response to salt stress is defective. Furthermore, molecular profiling also showed that DRB1 and DRB2 are both required for miRNA production during salt stress, and that both a target transcript cleavage mode and a translational repression mode of RNA silencing are required to appropriately regulate miRNA target gene expression as part of the molecular response of *Arabidopsis* to salt stress. Taken together, the phenotypic, physiological, and molecular analyses performed here clearly show that all components of the miRNA pathway must be fully functional for *Arabidopsis* to mount an appropriate miRNA-mediated molecular response to salt stress.

## 1. Introduction

The frequency and intensity of periods of abiotic stress has increased in recent decades either as a direct or indirect result of anthropogenically driven climate change. This alarming trend is ultimately reducing the global capability to produce the ‘viable’ crop volume required to provide food security, while in parallel, producing an adequate supply stock to be used as an alternative and sustainable biofuel for a rapidly expanding world population [1,2,3,4,5]. One abiotic stress which has become highly pronounced in recent decades is salinity, and now globally, over 800 million hectares of arable soils are impacted by this abiotic stress in its various forms, including groundwater-associated salinity, transient salinity, and irrigation-related salinity [6].

In addition to their central role in regulating developmental gene expression, microRNAs (miRNAs), and miRNA-directed gene expression regulation also function as important regulatory molecules central to the molecular response of a plant to abiotic stress [3,7,8]. Alterations to either the level of accumulation of an individual miRNA or the expression of its specific target gene(s) has been described in the genetic model plant species *Arabidopsis thaliana* (*Arabidopsis*) post the exposure of *Arabidopsis* to abiotic stimuli such as salt stress [9,10,11]. For example, Song et al. [12] showed that either the overexpression of miR394 or the knocking out of the expression of its target gene, *LEAF CURLING RESPONSIVENESS* (*LCR*), rendered the resulting *Arabidopsis* lines hypersensitive to salt stress. The authors went on to show that the altered salt stress response of the generated lines stemmed from modified root system architecture in both the miR394 overexpression and *LCR* knockout plant lines. Similarly, we have shown that the overexpression of miR397 from the C_4_ monocotyledonous grass *Setaria viridis*, in *Arabidopsis*, increased the sensitivity of the generated transformant lines to salt stress compared to unmodified wild-type *Arabidopsis* plants [13]. Furthermore, we went on to show that the degree of sensitivity to the imposed salt stress treatment regime was tightly correlated with the extent to which shoot and root development was defective in individual transformant lines, and these plant development defects were, in turn, associated with the level of *S. viridis* miR397 over-accumulation [13]. Considering the central role occupied by miRNA-mediated molecular responses to salt stress, it is unsurprising that the focus of contemporary research is aimed at constructing a more detailed understanding of the fundamental, abiotic stress-induced miRNA-mediated adaptive response networks in plants.

Of the five members of the *Arabidopsis* DOUBLE-STRANDED RNA BINDING (DRB) protein family, DRB1 and DRB2 have been assigned central functional roles in the production stage of the *Arabidopsis* miRNA pathway [14,15,16,17,18,19,20]. More specifically, DRB1, via its functional partnership with the DICER-LIKE1 (DCL1) endonuclease, is required to produce most *Arabidopsis* miRNAs [14,15,16]. In contrast to DRB1, DRB2 only appears to form a functional interaction with DCL1 to produce a specific cohort of *Arabidopsis* miRNAs in the tissues where *DRB2* expression overlaps with the expression of *DRB1* [17,18,19]. In addition, previous research has indicated that DRB1 and DRB2 act as molecular switches to determine whether a specific *Arabidopsis* miRNA directs the canonical mode of miRNA-directed RNA silencing in plants, target messenger RNA (mRNA) cleavage, or via the alternate mode of miRNA-directed RNA silencing, translational repression, to regulate the expression of its target gene(s) [17,18,19,20]. It has also been suggested that either the DRB1-dependent or DRB2-dependent miRNA pathway may have diverged from the other during plant genome evolution to allow DRB1-dependent miRNAs to primarily regulate the expression of developmentally important genes and for DRB2-dependent miRNAs to direct the expression regulation of those target genes involved in mounting a molecular response against abiotic and/or biotic stress [17,18,19,20].

To attempt to provide further evidence that the driving force for the divergence of a DRB2-dependent miRNA pathway from the predominant DRB1-dependent miRNA pathway in *Arabidopsis* was to provide a specialized form of a miRNA-mediated molecular response to abiotic stress, eight-day-old wild-type *Arabidopsis* plants (ecotype; Columbia-0 (Col-0)) and the *drb1* and *drb2* single mutants were exposed to a 7-day cultivation period in the presence of 150 millimolar (mM) sodium chloride (NaCl). The phenotypic analyses of fresh weight and rosette area, together with the physiological assessments of anthocyanin abundance and chlorophyll *a* content, identified the *drb2* mutant to be the most sensitive to the imposed stress regime. In addition, considerable differences in the molecular response of the three analyzed *Arabidopsis* lines were observed. Specifically, in salt-stressed Col-0 seedlings, 75% of miRNAs with altered abundance were increased in their levels, whereas in direct contrast, 81% and 65% of miRNAs with altered levels in salt-stressed *drb1* and *drb2* seedlings were reduced in their abundance. The opposing miRNA abundance trends documented for Col-0 seedlings, compared to *drb1* and *drb2* seedlings, readily showed that the ability of these two mutant lines to mount an appropriate miRNA-mediated molecular response to salt stress was defective. We further show that both DRB1 and DRB2 are required to efficiently regulate the production of the seven miRNAs selected for experimental validation, including miR160, miR164, miR167, miR396, miR399, miR408, and miR858. Finally, we show that both a target transcript cleavage mode and a translation repression mode of RNA silencing are required to provide an appropriate degree of control to regulate the expression of the target genes of the experimentally validated miRNAs during salt stress. In summary, although unsuccessful in our attempt to definitively show that a DRB2-dependent miRNA pathway has evolved from the central and developmentally crucial DRB1-dependent miRNA pathway to direct miRNA-mediated molecular responses to salt stress, we provide extensive evidence that the functional activity of both DRB1 and DRB2 is an essential requirement for *Arabidopsis* to mount an appropriate miRNA-mediated molecular response to salt stress.

## 2. Results

### 2.1. The Phenotypic and Physiological Assessment of 15-Day-Old Col-0, drb1, and drb2 Seedlings Following a 7-Day Salt Stress Treatment Regime

**Figure 1:**
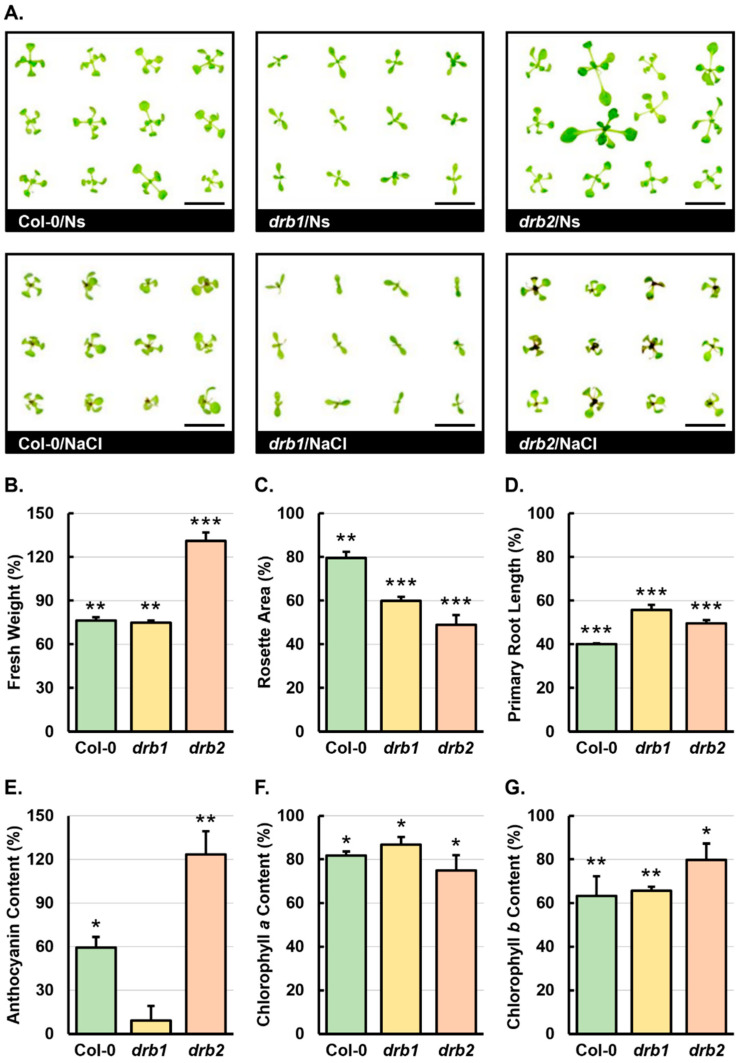
Phenotypic and physiological assessment of 15-day-old salt-stressed Col-0, *drb1*, and *drb2* seedlings. (**A**) Phenotypes expressed by 15-day-old control-grown Col-0/Ns, *drb1*/Ns, and *drb2*/Ns seedlings (top panel) and salt-stressed Col-0/NaCl, *drb1*/NaCl, and *drb2*/NaCl seedlings (bottom panels). Scale bar = 1.0 cm. (**B**–**G**) Each quantified metric of salt-stressed Col-0, *drb1*, and *drb2* seedlings was compared to those determined for non-stressed counterpart of each plant line. Differences in whole seedling fresh weight (**B**), rosette area (**C**), primarily root length (**D**), anthocyanin abundance (**E**), and chlorophyll *a* (**F**) and *b* (**G**) content were determined via assessment of four biological replicates, with each replicate consisting of a pool of 12 plants. Error bars represent standard deviation (±SD) and presence of an asterisk (*) represents statistically significant difference between salt stress and control samples (*p*-value: * < 0.05; ** < 0.005; *** < 0.001).

The differences in the development of the *drb1* and *drb2* single mutants compared to Col-0 plants at the same age have been described previously [15,17]. Figure 1A supports these previous findings showing the impeded development of non-stressed *drb1* (*drb1*/Ns) seedlings and the enhanced development of non-stressed *drb2* (*drb2*/Ns) seedlings compared to 15-day-old Col-0 seedlings cultivated under standard growth conditions (Col-0/Ns seedlings). Figure 1A also shows the ‘penetrance’ phenotype associated with the loss-of-function mutation harbored by the *drb2* mutant. More specifically, the *drb2* penetrance phenotype is characterized by a small proportion of seedlings (~10–15%) displaying highly promoted development, or vigor, compared to the more subtle promotion of the development displayed by most individuals in each *drb2* mutant plant population. The negative impact of a 7-day cultivation period on a growth medium supplemented with 150 mM NaCl on the development of Col-0, *drb1*, and *drb2* seedlings is also clearly depicted in Figure 1A, with Col-0/NaCl, *drb1*/NaCl, and *drb2*/NaCl plants displaying a reduction in overall size due to a combination of restricted developmental progression and the downward curling of rosettes leaves. Compared to their non-stressed counterparts, Col-0/NaCl, *drb1*/NaCl, and *drb2*/NaCl seedlings also displayed pigment alterations in their aerial tissues (Figure 1A), including chlorosis (Col-0/NaCl, *drb1*/NaCl, and *drb2*/NaCl plants) and anthocyanin accumulation in the region surrounding the shoot apical meristem (SAM) and extending into rosette leaf petioles (Col-0/NaCl and *drb2*/NaCl plants).

When compared to their non-stressed counterparts, 15-day-old Col-0/Ns and *drb1*/Ns seedlings, the fresh weight of Col-0/NaCl and *drb1*/NaCl whole seedlings was reduced by 23.7% and 25.1%, respectively (Figure 1B). In direct contrast, compared to *drb2*/Ns seedlings, the fresh weight of *drb2*/NaCl plants was determined to be significantly increased by 30.9% (Figure 1B). This formed an unexpected finding considering that a ‘stress’ phenotype was uniformly displayed by *drb2*/NaCl seedlings following their cultivation for 7 days on a NaCl supplemented medium (Figure 1A). The quantification of rosette area did, however, confirm the negative impact that the 7-day stress treatment period had on the developmental progression of Col-0/NaCl, *drb1*/NaCl, and *drb2*/NaCl plants, with the rosette area of these three *Arabidopsis* lines reduced by 20.5%, 40.1%, and 51.2%, respectively (Figure 1C). A negative impact on the development of 15-day-old Col-0, *drb1*, and *drb2* seedlings by the imposed stress was further evidenced by the 60.0%, 44.3%, and 50.4% reductions in the primary root length of Col-0/NaCl, *drb1*/NaCl, and *drb2*/NaCl plants, respectively (Figure 1D). The severe stress phenotype uniformly displayed by *drb2*/NaCl seedlings (Figure 1A) together with the significant reduction in the rosette area and primary root length of *drb2* seedlings following the 7-day salt stress treatment period (Figure 1C,D) further highlight that an increased fresh weight of *drb2*/NaCl whole seedlings, compared to *drb2*/Ns whole seedlings (Figure 1B), formed an unexpected finding stemming from the phenotypic characterization of the consequences of imposing a 7-day salt stress treatment regime on Col-0, *drb1*, and *drb2* development.

The accumulation of the flavonoid pigment, anthocyanin, is a well-documented plant defense mechanism to attempt to provide a degree of tolerance to abiotic stress [21,22,23]. The quantification of anthocyanin abundance revealed that the level of this flavonoid pigment was elevated by 59.3% and 123.3% in salt-stressed Col-0 and *drb2* plants, respectively (Figure 1E). However, in *drb1*/NaCl plants, anthocyanin abundance was only mildly increased by 9.2% when compared to the *drb1*/Ns sample (Figure 1E). Considering that photosynthesis is the fundamental pathway to fix carbon and to provide energy for plant growth and development, spectrophotometry was next used to quantify the levels of the two central photosynthetic pigments, chlorophyll *a* and *b* (Chl *a* and *b*). Furthermore, Chl *a* constitutes the primary photosynthetic pigment as it is responsible for converting photons to chemical energy via the light-dependent reactions of photosynthesis [24]. Spectrophotometry revealed the Chl *a* abundance to be significantly reduced in Col-0/NaCl, *drb1*/NaCl, and *drb2*/NaCl seedlings by 18.3%, 13.3%, and 25.1%, respectively, when compared to the non-stressed counterpart of each plant line (Figure 1F). While Chl *b* is not an essential pigment for photosynthesis, Chl *b* is an important auxiliary pigment, increasing the light absorption spectrum of a plant to allow for photosynthesis to continue under a broader range of light conditions [24]. Therefore, spectrophotometry was again used to quantify Chl *b* abundance and revealed that Chl *b* abundance was significantly reduced in salt-stressed Col-0, *drb1*, and *drb2* plants (Figure 1G). More specifically, compared to Col-0/Ns, *drb1*/Ns, and *drb2*/Ns seedlings, Chl *b* content was reduced by 36.7%, 34.4%, and 20.2% in Col-0/NaCl, *drb1*/NaCl, and *drb2*/NaCl plants (Figure 1G). Therefore, when considered together, the phenotypic (Figure 1B–D) and physiological analyses (Figure 1E–G) presented in Figure 1 readily confirm the severe negative impact that the 7-day salt stress treatment period had on the development of 15-day-old Col-0, *drb1*, and *drb2* seedlings.

### 2.2. The Molecular Profiling of the Gene Expression of the Core Protein Machinery of the miRNA Pathway in 15-Day-Old Col-0, drb1, and drb2 Seedlings Following a 7-Day Salt Stress Treatment Regime

**Figure 2:**
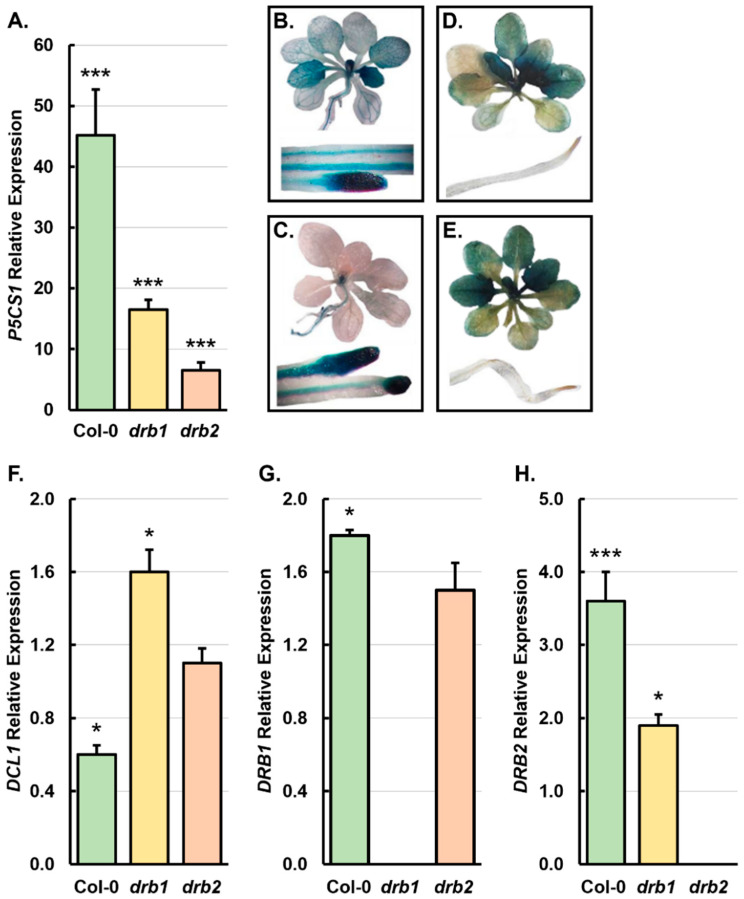
Molecular assessment of *P5CS1*, *DCL1*, *DRB1,* and *DRB2* gene expression in 15-day-old Col-0, *drb1*, and *drb2* seedlings following the 7-day salt stress treatment period. (**A**) RT-qPCR analysis of *P5CS1* expression in 15-day-old salt-stressed Col-0, *drb1*, and *drb2* seedlings. (**B**,**C**) Visualization of *GUS* reporter gene expression in the *DRB1pro-GUS* transformant line cultivated under standard *Arabidopsis* growth conditions (**B**) and following salt stress (**C**). (**D**,**E**) *GUS* expression in the *DRB2pro-GUS* transformant line cultivated under standard growth conditions (**D**) and following salt stress (**E**). (**F**–**H**) RT-qPCR assessment of *DCL1* (**F**), *DRB1* (**G**), and *DRB2* (**H**) gene expression in 15-day-old Col-0, *drb1* and *drb2* seedlings following salt stress. Error bars represent ±SD of four biological replicates with each replicate consisting of pool of 12 plants. The presence of an asterisk (*) represents a statistically significant difference between the salt stress sample and control sample (*p*-value: * < 0.05; *** < 0.001).

The well-characterized *Arabidopsis* stress-responsive gene, *P5CS1* (*AT2G39800*), encodes the Δ1-PYRROLINE-5-CARBOXYLATE SYNTHETASE (P5CS) protein which functions as the rate-limiting enzyme of the proline biosynthesis pathway [25,26]. Proline is a crucial amino acid, accumulating in plants in response to a range of abiotic stress stimuli due to its central role in scavenging free radicals and replenishing Nicotinamide Adenine Dinucleotide Phosphate (NADP^+^) levels [27,28]. It is therefore unsurprising that the expression of the *P5CS1* locus is induced in *Arabidopsis* in response to its exposure to a range of abiotic stresses, including salt stress [29]. Figure 2A clearly shows that the degree of expression of the stress-responsive gene, *P5CS*, was highly upregulated in 15-day-old salt-stressed Col-0, *drb1*, and *drb2* seedlings. More specifically, *P5CS1* transcript abundance was increased by 45.2-, 15.5-, and 6.5-fold in Col-0/NaCl, *drb1*/NaCl, and *drb2*/NaCl seedlings, respectively, compared to its levels of expression in Col-0/Ns, *drb1*/Ns, and *drb2*/Ns seedlings (Figure 2A).

A transgene-based approach was next used to determine the effect of the imposed stress on the transcriptional activity of *DRB1* and *DRB2* in *Arabidopsis* plants via the use of the putative promoter regions of the *DRB1* and *DRB2* genes to drive the expression of the *GUS* (*β-glucuronidase*) reporter gene following the introduction of the *DRB1pro-GUS* [15] and *DRB2pro-GUS* transgenes [17,19] into the Col-0 background. Figure 2B shows that, under standard growth conditions, *DRB1* is widely expressed in the aerial tissues of Col-0 plants with *GUS* expressed in emerging and newly emerged rosette leaves, and throughout the vasculature of mature rosette leaves of *DRB1pro-GUS* transformants. *GUS* was also expressed in the central vein of the primary root of *DRB1pro-GUS* plants, with its expression concentrated in the tips of lateral roots and the primary root tip of *DRB1pro-GUS* plants (Figure 2B). Following the 7-day salt stress treatment period, *GUS* expression became tightly restricted to the SAM region in the aerial tissues of the *DRB1pro-GUS* transformant line (Figure 2C). In direct contrast, the expression of the reporter gene in *DRB1pro-GUS* roots remained largely unchanged by the imposed stress (Figure 2B,C). In the aerial tissues of non-stressed *DRB2pro-GUS* plants, *GUS* was expressed widely throughout the blade of both young and mature rosette leaves (Figure 2D). However, *GUS* was not expressed in the root system of *DRB2pro-GUS* plants when this transformant line was cultivated under standard *Arabidopsis* growth conditions (Figure 2D). The level and pattern of *GUS* expression in both the aerial and root tissues of the *DRB2pro-GUS* transformation line remained unchanged following the 7-day salt stress treatment period (Figure 2E). When considered together, the reporter gene expression data (Figure 2B–E) readily demonstrated that the *DRB1* gene is considerably more responsive to salt stress than the *DRB2* gene. However, the dramatic change to the level of *GUS* expression observed in salt-stressed *DRB1pro-GUS* plants was restricted to the aerial tissues of this transformant line.

The requirement of the DCL1/DRB1 functional partnership to produce most of the miRNA sRNAs which accumulate in *Arabidopsis* is well documented [30,31,32]. The involvement of DRB2, most likely via the formation of a functional partnership with DCL1 to produce specific miRNA cohorts in tissues where *DRB2* is expressed in wild-type *Arabidopsis* plants, has also been demonstrated [17,18,19]. Therefore, a standard reverse transcriptase quantitative PCR (RT-qPCR) approach was applied to document any change to the level of expression of the genes which encode these three core pieces of the protein machinery of the production stage of the *Arabidopsis* miRNA pathway in 15-day-old Col-0, *drb1*, and *drb2* seedlings following salt stress (Figure 2F–H). The expression of *DCL1* was reduced by 1.7-fold in Col-0/NaCl seedlings compared to its level of expression in Col-0/Ns seedlings. In contrast, *DCL1* transcript abundance was significantly elevated by 1.6-fold in salt-stressed *drb1* seedlings and only mildly increased by 1.2-fold in *drb2*/NaCl plants compared to the level of *DCL1* gene expression in *drb1*/Ns and *drb2*/Ns plants, respectively (Figure 2F). Unsurprisingly, RT-qPCR failed to detect either *DRB1* or *DRB2* expression in the corresponding *drb1* and *drb2* mutant backgrounds (Figure 2G,H). However, this analysis did reveal that *DRB1* expression was significantly elevated by 1.8-fold in Col-0/NaCl plants and moderately increased by 1.5-fold in *drb2*/NaCl plants when compared to the level of *DRB1* expression in Col-0/Ns and *drb2*/Ns plants, respectively (Figure 2G). In salt-stressed Col-0 and *drb1* seedlings, *DRB2* expression was significantly elevated by 3.6- and 1.9-fold, respectively (Figure 2H). Therefore, when considered together, RT-qPCR indicated that the imposed 7-day salt stress treatment period largely had a positive influence on the transcriptional activity of the three analyzed genes which encode core pieces of the protein machinery of the *Arabidopsis* miRNA pathway (Figure 2F–H).

### 2.3. Profiling of miRNA Landscapes of Salt-Stressed Col-0, drb1, and drb2 Seedlings via Small RNA Sequencing and Experimental Analysis of miRNA Abundance via RT-qPCR

**Figure 3:**
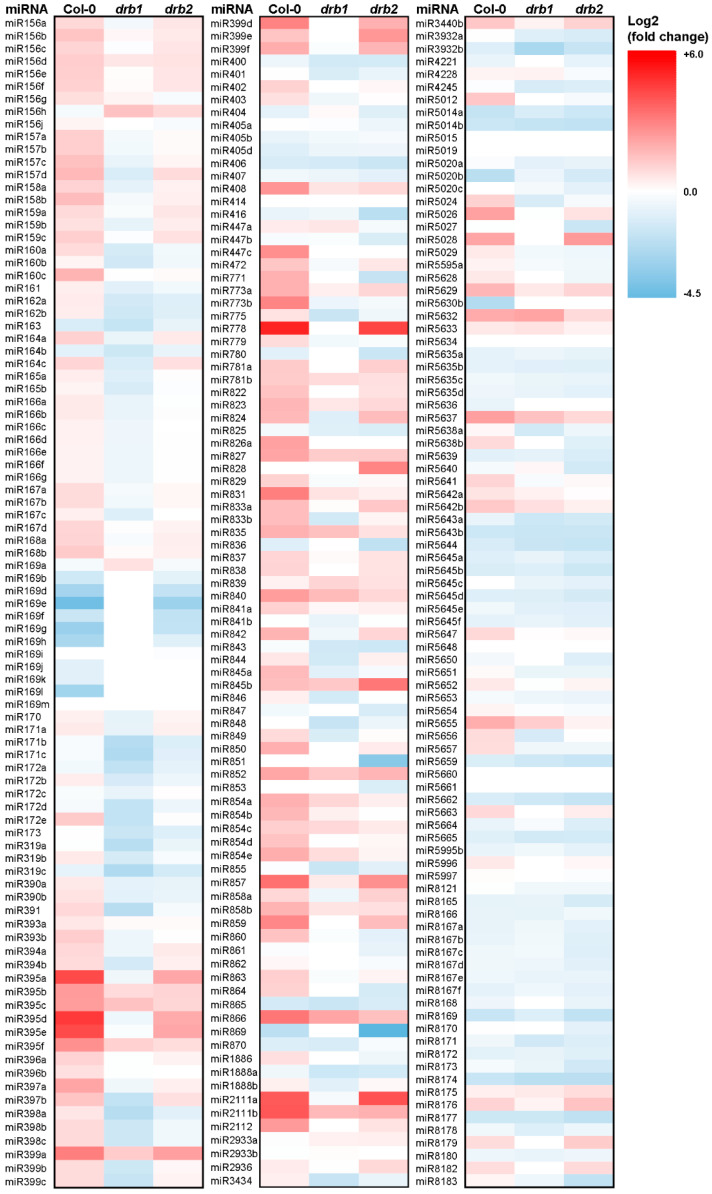
Molecular profiling of miRNA abundance by sRNA-Seq to establish miRNA landscapes of 15-day-old Col-0, *drb1*, and *drb2* seedlings following the 7-day salt stress treatment period. The sRNA-Seq approach was used to establish the degree of alteration to miRNA landscapes of 15-day-old Col-0, *drb1*, and *drb2* whole seedlings following a 7-day cultivation period of 8-day-old seedlings on growth medium supplemented with 150 mM NaCl. Per vertical column of the heatmap, each individual tile represents a single miRNA, and the intensity of red-colored shading indicates the degree of miRNA abundance upregulation, while the intensity of the blue-colored shading represents the extent of accumulation downregulation for each individual miRNA.

Considering that RT-qPCR revealed the expression of *DCL1*, *DRB1*, and *DRB2* to be altered by the imposed stress (Figure 2F–H), small RNA sequencing (sRNA-Seq) was next employed to establish the extent of alteration to the miRNA populations of 15-day-old salt-stressed Col-0, *drb1*, and *drb2* whole seedlings. Initially, sequencing of the miRNA population of control-grown Col-0, *drb1*, and *drb2* whole seedlings detected near equivalent miRNA numbers in the Col-0/Ns and *drb2*/Ns samples, with 262 and 258 miRNAs identified, respectively. In control-grown *drb1* whole seedlings, however, the total number of miRNAs detected by sRNA-Seq was reduced to 221. Considering that DRB1 is required to assist DCL1 in the production of most *Arabidopsis* miRNAs [30,31,32], the detection of a reduced number of miRNAs in the *drb1*/Ns sample, compared to the Col-0/Ns and *drb2*/Ns samples, formed an expected result. Figure 3 shows the widespread alteration to the abundance of many of the miRNAs detected as part of the total miRNA populations of salt-stressed Col-0, *drb1*, and *drb2* seedlings. More specifically, the comparison of miRNA abundance in the salt-stressed samples, compared to the profiles established by sRNA-Seq analysis of control-grown Col-0, *drb1*, and *drb2* whole seedlings, revealed that the abundance of 118, 82, and 83 miRNAs was significantly altered (either elevated or reduced) in Col-0/NaCl, *drb1*/NaCl, and *drb2*/NaCl seedlings, respectively. Therefore, of the total number of miRNAs detected in non-stressed Col-0, *drb1*, and *drb2* plants, salt stress significantly altered the abundance of 45.0%, 37.1%, and 32.2% of the global miRNA populations of these three *Arabidopsis* lines. Interestingly, a higher degree of abundance alteration in salt-stressed Col-0 plants, than in either the *drb1*/NaCl or *drb2*/NaCl sample, further suggests that not only does the loss of DRB1 or DRB2 function negatively influence the efficiency of miRNA production, but that both loss-of-function mutations also appear to adversely impact the ability of *Arabidopsis* to mount an appropriate miRNA-directed molecular response to salt stress.

The profiling of miRNAs with significantly altered abundance in the Col-0/NaCl, *drb1*/NaCl, and *drb2*/NaCl samples also shows that the imposed stress had a different influence on the global miRNA populations of Col-0 plants, compared to the general trend of influence the imposed stress had on the miRNA populations of *drb1*/NaCl and *drb2*/NaCl plants (Figure 3). Moreover, in Col-0/NaCl seedlings a general trend of promoted miRNA accumulation was observed, with 89 of the 118 significantly altered miRNAs (75.4%) increased in abundance in Col-0/NaCl plants compared to their abundance in the Col-0/Ns sample. In contrast, Figure 3 clearly shows the general downward trend in miRNA abundance in the salt-stressed *drb1* and *drb2* samples when compared to their respective control-grown counterparts. This downward trend in miRNA abundance was more pronounced in the *drb1* mutant background than it was in the *drb2* mutant post the application of stress, with 80.5% (n = 66/82) of the miRNAs with a significantly altered abundance having a reduced level of accumulation in the *drb1*/NaCl sample. In comparison, only 65.1% (n = 54/83) of the miRNAs with significantly altered abundance post the application of salt stress had a reduced level of accumulation in the *drb2* mutant background. When considered together, the opposing trends in miRNA abundance alteration in Col-0 seedlings, compared to *drb1* and *drb2* plants, indicated that the miRNA-mediated molecular response to salt stress was defective in the absence of either DRB1 or DRB2 function.

The sRNA-Seq approach employed here to profile miRNA abundance changes in 15-day-old salt-stressed Col-0, *drb1*, and *drb2* plants clearly showed that the abundance of many of the miRNA species which accumulate in *Arabidopsis* whole seedlings was altered by the imposed stress (Figure 3). Therefore, RT-qPCR was next applied to experimentally analyze the miRNA accumulation trends identified by sRNA-Seq with members of seven *MICRORNA* (*MIR*) gene families selected for this analysis. Moreover, RT-qPCR confirmation of the sRNA-Seq-identified abundance trends was attempted for the *MIR160*, *MIR164*, *MIR167*, *MIR396*, *MIR399*, *MIR408,* and *MIR858* gene families. Figure S1A shows that most members of the *MIR160*, *MIR164*, *MIR167,* and *MIR396* gene families were decreased in abundance in control-grown *drb1* seedlings and had increased accumulation in the *drb2*/Ns sample. DRB2 has been shown to be antagonistic towards the action of DRB1 in its formation of a functional partnership with DCL1 to produce specific cohorts of miRNAs in *Arabidopsis* tissues where *DRB2* expression overlaps with the expressional domain of *DRB1*, including the SAM region in aerial tissues and the root apical meristem (RAM) in the root system of *Arabidopsis* plants (Figure 2B–E) [17,18,19]. Therefore, members of these four *MIR* gene families were selected for inclusion in the RT-qPCR analyses to determine if the interplay of DRB1 and DRB2 in the production of these miRNAs had any influence on the sRNA-Seq-identified miRNA accumulation trends in salt-stressed *drb1* and *drb2* seedlings.

Members of the *MIR399* and *MIR858* gene families were revealed by sRNA-Seq to exhibit similar abundance trends in control-grown *drb1* and *drb2* seedlings (Figure S1A), which potentially indicates that both DRB1 and DRB2 are required to produce these two miRNAs. We have shown previously [17,18] that DRB2 can also function in a synergistic manner to DRB1 in the DCL1/DRB1 functional partnership to produce a specific cohort of miRNAs in *Arabidopsis* tissues where *DRB1* and *DRB2* expression overlaps (i.e., the SAM and RAM). The abundance of miR408 was also included as a candidate for RT-qPCR analysis as sRNA-Seq revealed the miR408 abundance to remain unchanged and to be elevated in *drb1*/Ns and *drb2*/Ns seedlings, respectively, when compared to its accumulation level in Col-0/Ns seedlings (Figure S1A). This distinct abundance profile indicated that DRB2 may play a more prominent role in miR408 production in *Arabidopsis* seedlings than DRB1. Therefore, together, the abundance of members of these three *MIR* gene families were also quantified via RT-qPCR as part of the experimental analysis of the sRNA-Seq data to determine what influence, if any, the DRB1/DRB2 functional interplay had on the accumulation of the miRNAs miR399, miR408, and miR858 in salt-stressed *drb1* and *drb2* seedlings. Finally, the accumulation level of individual *MIR* gene family members was summed together to identify any shared abundance trends across the seven miRNAs selected for further analysis in non-stressed *drb1* and *drb2* whole seedlings for comparison to the Col-0/Ns sample. This approach revealed that, compared to the Col-0/Ns sample, the levels of miR160, miR164, miR167, miR396, and miR399 were reduced in *drb1*/Ns seedlings (Figure S1B). In contrast, the miR408 abundance remained largely unchanged, and miR858 levels were mildly increased in *drb1*/Ns seedlings, when compared to the Col-0/Ns sample. Of the seven miRNAs selected for further analysis, only the level of miR399 was reduced in *drb2*/Ns whole seedlings, with the abundance of miR160, miR164, miR167, miR396, miR408, and miR858 elevated to different degrees in this mutant background compared to the Col-0/Ns sample (Figure S1B).

**Figure 4:**
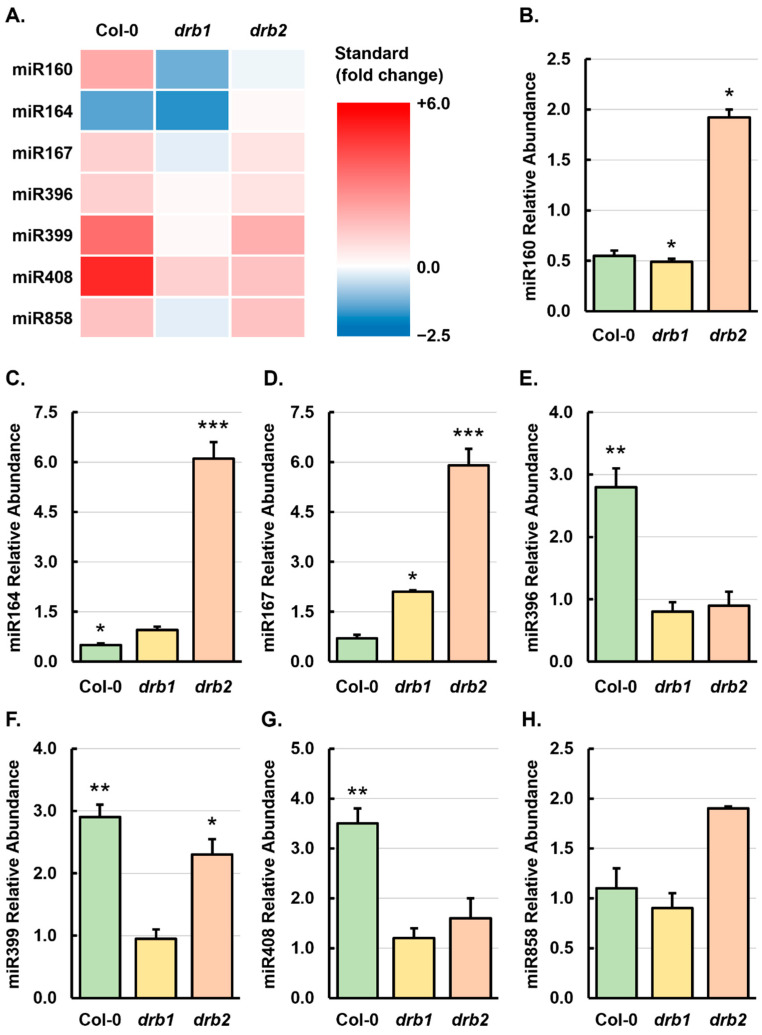
Profiling of miRNA accumulation in 15-day-old Col-0, *drb1*, and *drb2* seedlings by sRNA-Seq and RT-qPCR following salt stress. (**A**) Profiling by sRNA-Seq of abundance trends of the *MIR160*, *MIR164*, *MIR167*, *MIRR396*, *MIR399*, *MIR408,* and *MIR858* gene families post summing together abundance trends of individual family members in 15-day-old salt-stressed Col-0, *drb1*, and *drb2* seedlings. Shading intensity (light to dark) of each tile of each column depicts the degree of abundance change presented as a standard fold change. (**B**–**H**) RT-qPCR quantification of the abundance of miRNAs miR160 (**B**), miR164 (**C**), miR167 (**D**), miR396 (**E**), miR399 (**F**), miR408 (**G**), and miR858 (**H**) in 15-day-old Col-0, *drb1*, and *drb2* seedlings following salt stress with miRNA abundance compared to the non-stressed counterpart of each *Arabidopsis* line. Error bars represent ±SD of four biological replicates with each replicate consisting of a pool of 12 plants. The presence of an asterisk (*) above a column represents a statistically significant difference between the salt stress and control samples (*p*-value: * < 0.05; ** < 0.005; *** < 0.001).

In a previous *Arabidopsis* study where the precursor transcript of miR160, *PRE-MIR160A*, was overexpressed, it was shown that the resulting seedlings had reduced sensitivity to abscisic acid (ABA) stress [33], with ABA forming a key hormone in directing the physiological responses of a plant to abiotic stress, including salt stress. Furthermore, miR160 has been shown to be responsive to salt stress in several other plant species including *Setaria* (*Setaria viridis*) [34], peanut (*Arachis hypogaea*) [35], and Ginkgo (*Ginkgo biloba*) [36]. Figure 3 shows that the miR160a, miR160b, and miR160c abundance was elevated in Col-0/NaCl plants compared to the abundance of these three *MIR160* gene family members in non-stressed Col-0 plants. In contrast, sRNA-Seq indicated that the abundance of all three *MIR160* family members was reduced in salt-stressed *drb1* and *drb2* seedlings compared to the level of miR160a, miR160b, and miR160c accumulation in *drb1*/Ns and *drb2*/Ns seedlings (Figure 3). Elevated (up 2.3-fold), mildly reduced (down 1.3-fold), and largely unchanged abundance trends were identified for miR160 when the levels of all three family members were summed together in salt-stressed Col-0, *drb1*, and *drb2* seedlings, respectively (Figure 4A) (Table S1). However, quantification of miR160 abundance via RT-qPCR revealed opposing trends for miR160 abundance in 15-day-old Col-0, *drb1* and *drb2* seedlings following the 7-day cultivation period on *Arabidopsis* growth medium supplemented with 150 mM NaCl. More specifically, RT-qPCR showed that the level of miR160 accumulation was reduced by 1.8- and 2.0-fold in salt-stressed Col-0 and *drb1* seedlings, respectively, and elevated by 1.9-fold in *drb2*/NaCl seedlings (Figure 4B).

The miR164 sRNA has been reported to be responsive to salt stress in the crop species wheat (*Triticum aestivum*) [37], rice (*Oryza sativa*) [38], and maize (*Zea mays*) [39], and in the invasive weed species saltmarsh cordgrass (*Spartina alterniflora*) [40]. However, to date, miR164 has not been specifically reported as a salt stress-responsive miRNA in *Arabidopsis*. Interestingly, sRNA-Seq revealed miR164a, miR164b, and miR164c abundance to be altered in all three assessed *Arabidopsis* lines post salt stress (Figure 3). Namely, miR164a and miR164c levels were increased in salt-stressed Col-0 and *drb2* seedlings and reduced in the *drb1*/NaCl sample, and the level of miR164b was determined to be reduced in all three salt-stressed samples (Figure 3). Interestingly, due to the higher levels of miR164b abundance, compared to the abundance of miR164a and miR164c, whole family abundance analysis (Figure 4A) revealed miR164 to be reduced by 1.4- and 2.3-fold in salt-stressed Col-0 and *drb1* seedlings, respectively, and to accumulate to a highly similar level in the *drb2*/Ns (1032 reads) and *drb2*/NaCl (1081 reads) samples (Table S1). The quantification of miR160 abundance by RT-qPCR confirmed that miR164 levels were reduced in salt-stressed Col-0 seedlings. However, this analysis also indicated that the miR164 abundance remained largely unchanged in *drb1*/NaCl plants and was significantly elevated by 6.1-fold in *drb2*/NaCl seedlings (Figure 4C).

Via the use of microarray and RT-PCR technologies, Liu et al. [41] showed that in *Arabidopsis*, miR167 is responsive to salt stress (300 mM NaCl). Similarly, a salt stress-induced alteration to the abundance of miR167 has also been reported in *Setaria* [34], rice [38], maize [39], salt cedar (*Tamarix chinensis*) [42], and tomato (*Solanum lycopersicum*) [43]. The sRNA-Seq analysis conducted here revealed that the abundance of all four members of the *Arabidopsis MIR167* family was elevated in Col-0/NaCl plants (Figure 3). In salt-stressed *drb2* seedlings, the abundance of all four members of the *Arabidopsis MIR167* gene family was also increased compared to their accumulation level in *drb2*/Ns plants; however, the degree of the enhancement to miR167 levels was much milder in the *drb2* mutant compared to that determined for salt-stressed Col-0 seedlings (Figure 3). As revealed for most miRNAs in the salt-stressed *drb1* sample (Figure 3), miR167a, miR167b, miR167c, and miR167d abundance was determined to be reduced in the *drb1*/NaCl sample. Summing together the read counts for all four *MIR167* gene family members (Table S1) similarly showed that miR167 abundance was elevated by 1.7- and 1.3-fold in Col-0/NaCl and *drb2*/NaCl plants, respectively, and mildly reduced by 1.2-fold in the *drb1*/NaCl sample (Figure 4B). The quantification of the miR167 abundance in salt-stressed Col-0, *drb1*, and *drb2* plants by RT-qPCR (Figure 4D) did not align well with the miR167 profiles identified by sRNA-Seq (Figure 3 and Figure 4A). Namely, RT-qPCR indicated that miR167 abundance was mildly reduced by 1.4-fold in Col-0/NaCl plants, and was significantly elevated by 2.1- and 5.9-fold in *drb1*/NaCl and *drb2*/NaCl seedlings, respectively (Figure 4D).

The miR396 sRNA has been reported to be responsive to salt stress in a wide range of plant species including rice [38,44], *Setaria* [34], cotton (*Gossypium hirsutum*) [45,46], alfalfa (*Medicago truncatula*) [47], creeping bentgrass (*Agrostis stolonifera*) [48], and orange daylily (*Hemerocallis falva*) [49]. In addition, we have shown that the molecular manipulation of the miR396 expression module in *Arabidopsis* alters the response of *Arabidopsis* to salt stress [50]. It was therefore unsurprising that sRNA-Seq showed miR396a and miR396b abundance to be elevated in Col-0/NaCl seedlings and to be reduced in *drb1*/NaCl seedlings (Figure 3). In salt-stressed *drb2* plants, the abundance of the miR396a sRNA was mildly elevated while the accumulation level of miR396b showed a similar degree of mildly reduced abundance (Figure 3). Interestingly, due to miR396b being more abundant than miR396a, when miR396 abundance was considered as a single entity (Table S1), its abundance was revealed to be elevated by salt stress in all three *Arabidopsis* backgrounds (Figure 4A). Although RT-qPCR confirmed miR396 abundance to be elevated in Col-0/NaCl seedlings (up by 2.8-fold), this assessment approach alternatively indicated miR396 abundance to be mildly reduced by 1.3- and 1.1-fold in *drb1*/NaCl and *drb2*/NaCl seedlings, respectively (Figure 4F).

In *Arabidopsis*, miR399 and its target gene *PHOSPHATE2* (*PHO2*) are central to its response to phosphate (PO_4_) stress [51,52], and via the use of a molecular manipulation approach, we have shown that the miR399/*PHO2* expression module also plays a role in the adaptive response of *Arabidopsis* to salt stress [53]. Furthermore, we have also shown that DRB1 and DRB2 both have direct regulatory roles in controlling the miR399/*PHO2* expression module [54]. In 15-day-old salt-stressed Col-0 seedlings, sRNA-Seq indicated that the abundance of all detected *MIR399* gene family members was elevated, an altered miR399 abundance trend which was also documented in *drb2*/NaCl seedlings. In the *drb1* mutant background, the accumulation level of each *MIR399* gene family member was decreased by salt stress (Figure 3), except for the abundance of miR399a which was increased in *drb1*/NaCl plants. When the abundance of detected *MIR399* gene family members was summed together (Figure 4A) (Table S1), the accumulation of miR399 was determined to be elevated by 4.0-, 1.1-, and 2.5-fold in Col-0/NaCl, *drb1*/NaCl, and *drb2*/NaCl seedlings, respectively. RT-qPCR confirmed miR399 accumulation to be significantly elevated by 2.9- and 2.3-fold in salt-stressed Col-0 and *drb2* seedlings, respectively (Figure 4F), as well as indicated that miR399 levels remained largely unchanged between the *drb1*/Ns and *drb1*/NaCl samples.

To date, a role for miR408 in the response of a plant to salt stress has been demonstrated for *Salvia miltiorrhiz* (Chinese sage) via the constitutive expression of the *Smi*-miR408 precursor sequence in *Nicotiana benthamiana* [55]. In addition, we have previously experimentally verified that miR408 is a salt stress-responsive miRNA in *Setaria* [34]. Although miR408 has not yet been established as a salt stress-responsive miRNA in *Arabidopsis*, an altered miR408 abundance in *Arabidopsis* tissues has been reported for other abiotic stresses, including drought [56], sucrose [57], copper [58], sulfur [59], and temperature [60] stress, which together readily identify miR408 as an abiotic stress-responsive miRNA in *Arabidopsis*. Figure 4A shows that sRNA-Seq also identified miR408 as a salt stress-responsive miRNA with the abundance of miR408 elevated in 15-day-old salt-stressed Col-0, *drb1,* and *drb2* seedlings. The salt stress promotion of miR408 accumulation in *Arabidopsis* tissues was confirmed by RT-qPCR, which revealed miR408 levels to be significantly elevated by 3.5-fold in Col-0/NaCl plants and to be elevated to a lesser degree (up 1.2- and 1.6-fold, respectively) in the *drb1*/NaCl and *drb2*/NaCl samples (Figure 4G).

Via the use of high throughout sequencing approaches, miR858 has been demonstrated to be responsive to light alterations and to chilling, cadmium, and drought stress in green apple (*Malus domestica* ‘Granny Smith’) [61], watermelon (*Citrullus lanatus*) [62], rapeseed (*Brassica napus*) [63], and the medicinal plant species *Ammopiptanthus mongolicus* [64], respectively. The high throughput sequencing approach applied here suggested that in *Arabidopsis* at least, miR858 is also responsive to salt stress (Figure 3). Moreover, when the altered abundance of miR858a and miR858b was summed together, sRNA-Seq showed that miR858 abundance was elevated by 1.9-, 1.3-, and 2.3-fold (Table S1) in salt-stressed Col-0, *drb1,* and *drb2* plants, respectively (Figure 4A). RT-qPCR assessment of miR858 accumulation in salt-stressed Col-0, *drb1,* and *drb2* seedlings largely confirmed the sRNA-Seq-generated abundance profiles for miR858 (Figure 4H). Moreover, miR858 abundance was revealed by RT-qPCR to be mildly elevated by 1.1-fold in Col-0/NaCl seedlings, mildly reduced by the same degree in *drb1*/NaCl seedlings, and moderately promoted by 1.9-fold in *drb2*/NaCl seedlings (Figure 4H).

### 2.4. RT-qPCR Assessment of the Expression of the Target Genes of Arabidopsis miRNAs Demonstrated to Be Responsive to Salt Stress in 15-Day-Old Col-0, drb1, and drb2 Seedlings

**Figure 5:**
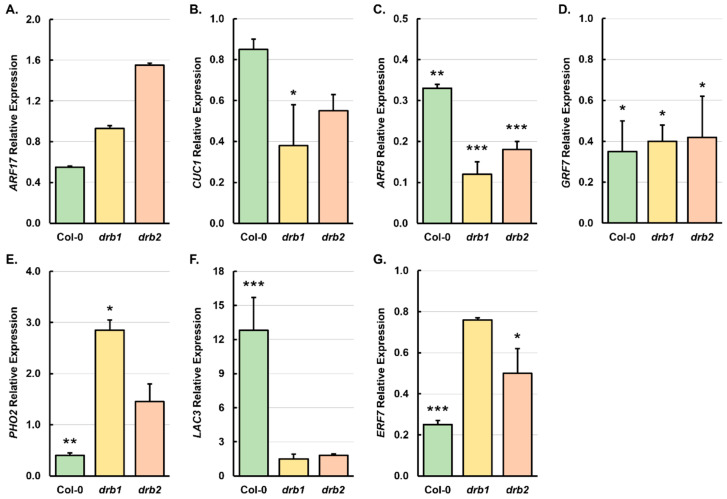
RT-qPCR assessment of miRNA target gene expression in 15-day-old *Col-0*, *drb1,* and *drb2* seedlings following salt stress. (**A**–**G**) RT-qPCR quantification of *ARF17* (**A**), *CUC1* (**B**), *ARF8* (**C**), *GRF7* (**D**), *PHO2* (**E**), *LAC3* (**F**), and *ERF7* (**G**) transcript abundance, respective target genes of miR160, miR164, miR167, miR396, miR399, miR408, and miR858, in 15-day-old Col-0, *drb1,* and *drb2* seedlings following 7-day salt stress treatment period. Target gene expression (presented as standard fold change) in each salt-stressed plant line was determined via comparison to non-stressed control-grown counterpart of each line. Error bars represent ±SD of four biological replicates with each biological replicate consisting of pool of 12 plants. Presence of asterisk (*) above column represents statistically significant difference between salt stress sample and control sample (*p*-value: * < 0.05; ** < 0.005; *** < 0.001).

Next, RT-qPCR was applied to correlate any changes to the expression of the target genes of the seven miRNAs shown to be responsive to salt stress. In *Arabidopsis*, miR160 regulates the expression of three closely related members of the *AUXIN RESPONSE FACTOR* (*ARF*) gene family of transcription factors, including *ARF10*, *ARF16,* and *ARF17* [65,66]. Figure 5A shows that the abundance of the miR160 target gene *ARF17* was reduced by 1.8- and 1.1-fold in salt-stressed Col-0 and *drb1* seedlings, respectively. In contrast, in 15-day-old *drb2*/NaCl plants, *ARF17* expression was promoted 1.6-fold (Figure 5A). Considering that miR160 has been shown to regulate *ARF17* expression via a transcript cleavage-based mechanism of RNA silencing [65], a shared miR160 and *ARF17* abundance profile in Col-0/NaCl, *drb1*/NaCl, and *drb2*/NaCl seedlings (Figure 4B and Figure 5A) formed an unexpected result. In *Arabidopsis*, miR164 regulates the expression of members of a small subclade of the NAC-domain gene superfamily, which includes *CUP SHAPED COTYLEDON1* (*CUC1*) and *CUC2* [67,68]. In salt-stressed Col-0, *drb1,* and *drb2* seedlings, RT-qPCR showed that *CUC1* expression was reduced by 1.2-, 2.6-, and 1.9-fold, respectively (Figure 5B). Reduced *CUC1* expression was only expected for salt-stressed *drb2* plants considering that the imposed stress caused a significant 6.1-fold increase in the abundance of the targeting miRNA, miR164 (Figure 4C). Seeing as though sRNA-Seq indicated that miR164 abundance was reduced in the Col-0/NaCl and *drb1*/NaCl samples (Figure 4A) and that RT-qPCR analysis revealed elevated and unchanged miR164 abundance in salt-stressed Col-0 and *drb1* plants (Figure 4C), reduced *CUC1* expression in these two *Arabidopsis* lines (Figure 5B) again formed an unexpected result.

In *Arabidopsis*, the expression of *ARF6* and *ARF8* is regulated by miR167 with the miR167/*ARF6*/*ARF8* expression module demonstrated to be essential for multiple aspects of *Arabidopsis* development [69,70,71]. The 7-day salt stress treatment regime was shown by RT-qPCR to significantly repress *ARF8* gene expression by 3.3-, 8.2-, and 5.7-fold in Col-0, *drb1,* and *drb2* whole seedlings, respectively (Figure 5C). A repressed *ARF8* expression was expected in the salt-stressed *drb1* and *drb2* samples, with RT-qPCR showing miR167 levels to be significantly elevated by 2.1- and 5.9-fold, respectively, in these two mutant backgrounds (Figure 4D). The miR167 sRNA has previously been suggested to direct both a transcript cleavage and translational repression mode of RNA silencing to control the level of expression of its *ARF* target genes [70]. Therefore, the reduced abundance of *ARF8* and miR167 (down 1.4-fold) in salt-stressed Col-0 whole seedlings (Figure 4D and Figure 5C) may indicate that a translational repression mode of RNA silencing forms the predominant mode of the target gene expression regulation directed by miR167 under salt stress conditions. As shown for the miR167 target gene *ARF8*, the expression of the miR396 target gene, *GROWTH RESPONSE FACTOR7* (*GRF7*), was revealed by RT-qPCR to be significantly reduced by 2.8-, 2.5-, and 2.4-fold in salt-stressed Col-0, *drb1,* and *drb2* whole seedlings, respectively (Figure 5D). Repressed *GRF7* gene expression was expected in the Col-0/NaCl sample considering that the abundance of the targeting miRNA, miR396, was elevated 2.8-fold by the applied stress (Figure 4E). However, considering that RT-qPCR additionally showed that the accumulation level of miR396 was only mildly reduced by 1.3- and 1.1-fold in salt-stressed *drb1* and *drb2* seedlings, respectively (Figure 4E), a significantly repressed degree of *GRF7* target gene expression in these two mutant backgrounds again formed an unexpected result (Figure 5D).

*PHOSPHATE2* (*PHO2*) forms the single target gene for expression regulation at the posttranscriptional level for all six members of the *Arabidopsis MIR399* gene family [51,52]. Furthermore, we have shown previously that molecular manipulation of the miR399/*PHO2* expression modules alters the ability of *Arabidopsis* to response to salt stress [50]. In salt-stressed Col-0 seedlings, *PHO2* expression was reduced by 2.5-fold (Figure 5E) in response to the 2.9-fold elevation in the accumulation of the targeting miRNA, miR399 (Figure 4F). This finding suggests that in salt-stressed wild-type *Arabidopsis* plants, miR399 regulates the abundance of the *PHO2* transcript by a mRNA cleavage mode of RNA silencing. In salt-stressed *drb1* and *drb2* seedlings, *PHO2* expression was elevated by 2.9- and 1.5-fold, respectively (Figure 5E). The miR399 levels remained unchanged in salt-stressed *drb1* seedlings (Figure 4F), with the unchanged level of miR399 potentially failing to be able to regulate increased *PHO2* transcript abundance when the expression of the *PHO2* target gene was induced by the imposed stress. Similarly, although miR399 abundance was increased by 2.3-fold in salt-stressed *drb2* seedlings (Figure 4F), this increase in the targeting miRNA may not have reached a high enough level to continue to adequately control *PHO2* expression (Figure 5E) if the expression of this locus is highly induced by salt stress.

In *Arabidopsis*, miR408 and its target genes, including *LACCASE3* (*LAC3*), have been shown previously to be responsive to copper and iron stress [72,73]. Figure 5F shows that in response to the 7-day salt stress treatment regime imposed here, *LAC3* expression was significantly enhanced by 12.8-fold in Col-0/NaCl seedlings and only mildly increased by 1.5- and 1.8-fold in the *drb1*/NaCl and *drb2*/NaCl samples, respectively. Interestingly, RT-qPCR showed that the abundance of the *LAC3* targeting miRNA, miR408, was also induced by the imposed stress, with miR408 levels increased by 3.5-, 1.2-, and 1.6-fold in salt-stressed Col-0, *drb1,* and *drb2* seedlings (Figure 4G); an induction to miRNA abundance which was also revealed via sRNA-Seq (Figure 3 and Figure 4A). Increased miR408 and *LAC3* transcript abundance could result from the transcriptional activity of both the *MIR408* and *LAC3* loci being induced by the imposed stress, or the observed shared transcript abundance trend could result from the scaling of miR408 levels in accordance with that of its *LAC3* target transcript to attempt to control *LAC3* expression via a translational repression mode of miR408-directed RNA silencing. *ETHYLENE RESPONSE FACTOR7* (*ERF7*) encodes a member of the *ERF* subfamily of the *ERF*/*APETELLA2* gene family of transcription factors and forms a putative target gene of the recently evolved *Arabidopsis* miRNA, miR858 [74]. RT-qPCR revealed that salt stress repressed *ERF7* expression by 4.0-, 1.3-, and 2.0-fold in 15-day-old Col-0, *drb1,* and *drb2* seedlings, respectively (Figure 5G). In the Col-0/NaCl and *drb1*/NaCl samples, miR858 accumulation remained largely unchanged (up and down by 1.1-fold, respectively). However, in *drb2*/NaCl seedlings, miR858 abundance was elevated by 1.9-fold (Figure 4H), an abundance change that likely directed the 2.0-fold reduction in *ERF7* expression (Figure 5G) in this plant line following the application of salt stress.

## 3. Discussion

### 3.1. The Quantification of Phenotypic and Physiological Metrics in Salt-Stressed Col-0, drb1, and drb2 Seedlings Identified drb2 as the Arabidopsis Line Most Sensitive to the Imposed Stress

Of the six phenotypic and physiological metrics quantified in this study, whole seedling fresh weight, rosette surface area, anthocyanin abundance, and Chl *a* content, were altered to the greatest extent in the *drb2* mutant background (Figure 1). When considered together, these four distinct assessments all indicated that of the three *Arabidopsis* lines analyzed, *drb2* plants were the most sensitive to the imposed stress. In addition, the most striking finding stemming from these initial analyses was also identified in the *drb2* mutant. Specifically, although rosette surface area and primary root length were significantly reduced by 51.2% and 50.4%, respectively, in salt-stressed *drb2* seedlings (Figure 1C,D), the fresh weight of *drb2*/NaCl whole plants was significantly increased by 30.9% (Figure 1B), and not reduced, as was expected. An increase in plant size can result from either an increase in the size of cells or an increased number of cells being produced [75,76], with the latter accounting for the increased size of *drb2* plants compared to Col-0 plants at the same stage of vegetative development [15,16,17]. In an attempt to limit the deleterious effects of high Na^+^ ion concentration in the cell cytoplasm, a plant will sequester the excess Na^+^ ions via the compartmentalization of these into vacuoles by enhancing Na^+^/H^+^ antiporter activity; in addition to limiting or removing the excess Na^+^ ions via inhibiting the influx of additional Na^+^ ions into the roots and promoting the rate of Na^+^ ion efflux out of root cells, respectively [77,78]. Inhibiting the rate of transpiration via stomata closure also forms an avoidance mechanism of a plant experiencing salt stress, with a reduced rate of transpiration in turn ensuring that the elevated levels of Na^+^ ions taken up by the roots, are retained by the roots, to prohibit the over-accumulation of Na^+^ ions in aerial tissues [79,80]. Therefore, when considering these known phenotypic responses of a plant to growth in a high-salt environment, together with our findings reported here, it can be proposed that the increase in the whole seedling fresh weight of salt-stressed *drb2* seedlings may be due to a more rapid closure of the rosette leaf stomata, which could have occurred in parallel with increased levels of Na^+^ ions being sequestered into the vacuoles of the greater number of cells that form in *drb2* rosette leaves. In turn, Na^+^ ion compartmentalization by the vacuoles of *drb2* leaf cells may have led to increased levels of water molecules entering the vacuoles in a further attempt by the *drb2* mutant to minimize the degree of the over-accumulation of these potentially detrimental ions in this cell organelle.

The abundance of anthocyanin is elevated in numerous plant species post exposure to abiotic stress due to the ability of this flavonoid pigment to scavenge ROS, toxic biomolecules whose production is induced by abiotic stress, which would cause cellular injury if their level of accumulation is not negated [21,22,23]. In the *drb2* mutant background, the 7-day salt stress treatment period caused the abundance of anthocyanin to increase considerably by 123.3% (Figure 1E). By contrast, this represented more than a 2-fold increase in the degree to which anthocyanin over-accumulated in the Col-0/NaCl sample (59.3% increase) and a greater than 13-fold promotion in the level of anthocyanin measured in salt-stressed *drb1* seedlings (9.2% increase). The very large increase in anthocyanin abundance in salt-stressed *drb2* seedlings, together with the minor change to anthocyanin levels in salt-stressed *drb1* seedlings, strongly suggests that the appropriate level of regulation of the biosynthesis pathway of this flavonoid pigment is lost in both the *drb1* and *drb2* mutant backgrounds: mutants which harbor functional defects at the production stage of the *Arabidopsis* miRNA pathway. Moreover, the vast difference in the degree to which anthocyanin production was induced by the imposed stress in the *drb1* and *drb2* mutant backgrounds strongly indicated that a fully functional miRNA pathway forms an important component of the overall molecular-mediated response of *Arabidopsis* to salt stress.

The abundance of both the primary photosynthetic pigment, Chl *a* [24], and auxiliary photosynthetic pigment, Chl *b* [24], was reduced the least in salt-stressed *drb1* seedlings, compared to the greater degree of Chl *a* and *b* content reduction documented for Col-0/NaCl and *drb2*/NaCl plants (Figure 1F,G). Considering that DRB1 is the primary DRB protein required to form a functional partnership with DCL1 for the production of most *Arabidopsis* miRNAs [14,31,32,81,82], a more tempered response to the imposed salt stress treatment regime in the *drb1* mutant background may indicate that the miRNA-directed regulation of photosynthesis forms a crucial component of the overall molecular response of *Arabidopsis* to salt stress, and that the induction or activation of this required response mechanism is defective in the *drb1* mutant. Further support of this proposal, that the changes to Chl *a* and *b* abundance observed here may not be a direct result of the imposed stress, but are actually indicative of the requirement of a fully functional miRNA pathway to regulate photosynthesis during salt stress, is provided via the comparison of the level of change of these two photosynthetic pigments in Col-0 and *drb2* plants. More specifically, the Chl *a* abundance was reduced by 25.1% in *drb2*/NaCl seedlings, whereas the Chl *a* level was only reduced by 18.3% in the Col-0/NaCl sample (Figure 1F). In contrast, the Chl *b* content was decreased by a much higher degree (36.7%) in salt-stressed Col-0 seedlings than the documented reduction (20.2%) in the abundance of this auxiliary photosynthetic pigment in the *drb2*/NaCl sample (Figure 1G). As proposed for the *drb1* mutant, the contrasting Chl *a* and *b* abundance trends in salt-stressed Col-0 and *drb2* seedlings may again indicate that an appropriate level of regulatory complexity over the chlorophyll biosynthesis pathway is lost in the absence of DRB2 directing its documented role in the miRNA pathway [17,18,19]; hence, the ability of this mutant background to respond appropriately to growth in a high-salt environment is impeded. Furthermore, it is important to note here that in our previous analysis of the proteomes of the *drb1* and *drb2* single mutants, large and distinct gene cohorts relating to photosynthesis were identified [20]. Specifically, the abundance of 58 proteins with functional roles assigned to chloroplasts were revealed to be reduced in abundance in the *drb1* mutant background and in *drb2* plants; a distinct cohort of 23 proteins previously assigned roles in the ‘chlorophyll biosynthesis process’ were reduced in abundance [20]. Considered together, these findings could explain the differences in the physiological response of *drb1* and *drb2* plants to salt stress, as well as to identify the importance of the miRNA-directed regulation of photosynthesis as part of the overall response of *Arabidopsis* to salt stress.

### 3.2. The miRNA Landscapes of 15-Day-Old Salt-Stressed Col-0, drb1, and drb2 Seedlings Are Distinctly Altered

The level of expression of the stress response gene, *P5CS1* [29], was significantly induced by 45.2-fold by the imposed stress in Col-0/NaCl seedlings (Figure 2A). In comparison, a much milder expression response was documented in *drb1*/NaCl and *drb2*/NaCl seedlings, with the *P5CS1* expression increased by 15.5- and 6.5-fold, respectively (Figure 2A). Interestingly, as with certain assessed phenotypic and physiological metrics, this result suggests that in the absence of DRB1 or DRB2 function, *drb1* and *drb2* plants are compromised in their ability to mount a comprehensive molecular response, most likely a miRNA-mediated molecular response, to salt stress. If the difference in *P5CS1* expression induction in salt-stressed Col-0, *drb1,* and *drb2* seedlings is indeed indicating that the miRNA-mediated molecular response to salt stress is defective in both *drb* mutant backgrounds, then a lower level of *P5CS1* expression induction in *drb2*/NaCl seedlings, compared to *drb1*/NaCl seedlings, would further infer that the ability of the *drb2* mutant to mount an effective miRNA-mediated molecular response to salt stress is compromised to a greater degree in 15-day-old *drb2* seedlings than it is in the *drb1* mutant. This would form a highly interesting finding considering that, compared to the almost global requirement of DRB1 for miRNA production in *Arabidopsis*, DRB2 is only involved in producing specific miRNA cohorts [17,19], a role which is additionally restricted by the tissue-specific expression of the *DRB2* gene [15,17,18]. The idea that the ability to mount an appropriate miRNA-mediated molecular response to salt stress is more impaired in the *drb2* mutant than it is in either the Col-0 or *drb1 Arabidopsis* lines is additionally supported by our previous analysis of the proteome of this mutant with many of the proteins with significantly altered abundance in the *drb2* seedlings associated with molecular responses to (1) abiotic stimuli, (2) osmotic stress, and (3) salt stress [20]. This previous finding, together with the expression analysis of *P5CS1* presented in Figure 2A, and the specific ‘stress response’ phenotypic and/or physiological characteristics displayed by 15-day-old salt-stressed *drb2* seedlings (Figure 1) indicate that, of the three *Arabidopsis* lines analyzed in this study, the development of the *drb2* mutant was negatively impacted to the greatest degree by the imposed 7-day salt stress treatment regime.

The expression response of *DCL1* is also different in *drb1*/NaCl and *drb2*/NaCl plants, compared to Col-0/NaCl plants (Figure 2F). More specifically, RT-qPCR showed that *DCL1* expression was reduced by 1.7-fold in Col-0/NaCl seedlings; however, in contrast, the expression of *DCL1* was elevated by 1.7- and 1.2-fold in *drb1*/NaCl and *drb2*/NaCl seedlings, respectively (Figure 2F). Considering that DCL1 is the primary DCL endonuclease required for miRNA production in *Arabidopsis* [31,32], the opposing trend of altered *DCL1* expression in Col-0/NaCl seedlings, compared to salt-stressed *drb1* and *drb2* seedlings, again strongly indicated that the ability of *Arabidopsis* to mount an appropriate miRNA-mediated molecular response to salt stress is compromised in the absence of either DRB1 or DRB2 function. The difference in *DCL1* expression in salt-stressed Col-0, *drb1,* and *drb2* seedlings also indicated that the degree of alteration to the miRNA landscapes following the application of salt stress would be unique to each of the three assessed *Arabidopsis* lines. Indeed, an *Arabidopsis* line-specific alteration to the miRNA landscapes of salt-stressed Col-0, *drb1,* and *drb2* seedlings was observed with the variation in the level of responsiveness to the imposed stress readily demonstrated by the differences in the proportion of each of the total miRNA populations with a significantly altered abundance post the application of stress (Figure 3). More specifically, 45.0% (n = 118/262) of miRNAs detected in 15-day-old Col-0 whole seedlings were significantly altered in abundance by the imposed salt stress treatment regime. In contrast, only 37.1% (n = 82/221) and 32.2% (n = 83/258) of the miRNA species detected by sRNA-Seq in the *drb1*/Ns and *drb2*/Ns samples, respectively, were determined to have significantly altered abundance in the *drb1*/NaCl and *drb2*/NaCl samples. This result once again showed that the ability of *Arabidopsis* to mount an appropriate miRNA-directed molecular response to salt stress is impeded in the absence of the functional activity of either DRB1 or DRB2. Furthermore, the profiling of the miRNA landscapes of *drb1*/NaCl and *drb2*/NaCl plants, and comparison of these to that of the salt stress-induced miRNA landscape of Col-0/NaCl plants, additionally indicated that the ability of the *drb2* mutant to respond to salt stress is negatively impacted to a greater degree than it is in the *drb1* mutant background. The most striking feature identified by the sRNA-Seq profiling exercise was the difference in the general trend of response of miRNAs with altered abundance in Col-0 seedlings, compared to *drb1* and *drb2* seedlings. Namely, 75.4% (n = 89/119) of the miRNAs with significantly altered abundance in Col-0/NaCl seedlings were increased in abundance in response to the imposed stress. In direct contrast to this finding, 80.5% (n = 66/82) and 65.1% (n = 54/83) of the miRNAs with altered abundance in *drb1*/NaCl and *drb2*/NaCl plants, respectively, were reduced in abundance following the application of salt stress. This finding once again showed that the ability of the *drb1* and *drb2* mutants to mount an appropriate miRNA-mediated molecular response to salt stress is severely impeded in the absence of DRB1 and DRB2 function.

### 3.3. DRB1 and DRB2 Are Required for miRNA Production as Part of Standard Arabidopsis Development and During Salt Stress

The reduced levels of miR160, miR164, miR167, and miR396 abundance in the *drb1*/Ns sample (Figure S1A) showed that DRB1, together with DCL1, was the primary DRB protein responsible for producing these four miRNAs, as has been shown previously for most miRNAs which accumulate in *Arabidopsis* tissues [15,16,30,31,32]. In addition, the elevated level of accumulation in *drb2*/Ns seedlings of the miR160, miR164, miR167, and miR396 sRNAs when the abundance of all family members was summed together (Figure S1B) identified a secondary role for DRB2 in the production of the individual members of these four *MIR* gene families via DRB2 antagonism of the DRB1/DCL1 functional partnership [17,18,19]. In addition to being reduced in abundance in *drb1*/Ns plants, the abundance of *MIR399* gene family members, miR399a, miR399b, and miR399c, was also reduced in *drb2*/Ns seedlings (Figure S1A,B). The reduced abundance of these *MIR399* gene family members in both the non-stressed *drb1* and *drb2* samples confirmed our previous findings [17,18], which showed that DRB2 also possesses the ability to act in a synergistic manner to the function of DRB1 in the DRB1/DCL1 partnership to produce specific miRNAs in *Arabidopsis* tissues. When compared to Col-0/Ns seedlings, the unchanged and elevated abundance of miR408 in *drb1*/Ns and *drb2*/Ns seedlings (Figure S1A) indicated that, together with DCL1, DRB2 can compensate for the loss of DRB1 function for miR408 production. A central role for DRB2 in regulating the rate of miR408 production in *Arabidopsis* is further provided by our demonstration that the abundance of this miRNA is increased in the *drb2*/Ns sample (Figure S1). More specifically, this accumulation trend shows that in the absence of DRB2 antagonism, DRB1/DCL1 can more efficiently process the miR408 precursor transcript resulting in the elevated abundance of this miRNA in the *drb2* mutant background [17,18]. Figure S1 also shows that the abundance of miR858 was mildly elevated in both the *drb1*/Ns and *drb2*/Ns samples. This unique accumulation profile provides further evidence that the interplay between DRB1 and DRB2 adds an additional layer of regulatory complexity to control the rate of production of certain *Arabidopsis* miRNAs [17,18,19]. Moreover, the mildly elevated miR858 levels in both the *drb1*/Ns and *drb2*/Ns samples infer that in the absence of function of one of these two DRB proteins the other DRB protein can form a functional partnership with DCL1 for the continued production of the miR858 sRNA, at a near equivalent rate of production to that observed in wild-type *Arabidopsis* plants where both DRB1 and DRB2 are functional.

In salt-stressed *drb2* seedlings, RT-qPCR revealed miR160, miR164, and miR167 levels to be significantly elevated (Figure 4). In contrast, this analysis showed that the abundance of these three miRNAs was reduced in the Col-0/NaCl sample (Figure 4B–D). The opposing accumulation trends for miR160, miR164, and miR167 in Col-0/NaCl and *drb2*/NaCl seedlings not only confirmed the antagonistic action of DRB2 on the DRB1/DCL1 partnership to produce these three miRNAs, but demonstrated that the *drb2* mutant is dysfunctional in its ability to mount an appropriate miRNA-mediated molecular response to the imposed stress. The abundance of miR396 was significantly elevated by 2.8-fold in the Col-0/NaCl sample yet was only mildly reduced by 1.3- and 1.1-fold in the *drb1*/NaCl and *drb2*/NaCl, respectively (Figure 4E). The miR396 abundance trend in salt-stressed Col-0, *drb1,* and *drb2* samples indicates that DRB1 and DRB2 form a synergistic relationship with each other to function together with DCL1 to produce this miRNA during salt stress, and that further, the appropriate degree of regulatory control over miR396 production is lost in both mutant backgrounds. Synergism between the functional activity of DRB1 and DRB2 is also evident for miRNAs miR399 and miR408 during salt stress. The accumulation profiles constructed from the RT-qPCR analyses of these two miRNAs in Col-0/NaCl, *drb1*/NaCl, and *drb2*/NaCl seedlings (Figure 4F,G), further revealed that the ability of the miR399 and miR408 expression modules to respond appropriately to salt stress is also defective in the absence of DRB1 or DRB2 function. RT-qPCR indicated that miR858 abundance was only altered by any real degree in the salt-stressed *drb2* sample (up 1.9-fold) (Figure 4H). Elevated miR858 abundance in *drb2*/NaCl seedlings, compared to the largely unchanged abundance of this miRNA in Col-0/NaCl and *drb1*/NaCl, confirmed a central functional role for DRB2 in miR858 production (Figure S1B). More specifically, the unchanged miR858 abundance in the *drb1*/NaCl sample demonstrates that in the absence of DRB1 function, DRB2 can readily form a functional partnership with DCL1 to efficiently process the miR858 sRNA from its precursor transcripts, *PRE-MIR858A* and *PRE-MIR858B*.

### 3.4. DRB1-Dependent and DRB2-Dependent Mechanisms of Gene Expression Regulation Are Required to Control miRNA Target Gene Expression in Response to Salt Stress

In salt-stressed Col-0 seedlings, *ARF17*, *CUC1*, and *ARF8* expression was reduced by 1.8-, 1.2-, and 3.3-fold, respectively (Figure 5A–C). Considering that the abundance of the three targeting miRNAs, miR160, miR164, and miR167, was also shown by RT-qPCR to be reduced (Figure 4B–D), or to scale in accordance with the abundance of their respective target genes, these miRNA and target gene expression trends suggest that in 15-day-old wild-type *Arabidopsis* seedlings, a DRB2-dependent translational repression mode of RNA silencing forms the predominant mode of target gene expression regulation directed by these three miRNAs. A DRB2-dependent translational repression mode of RNA silencing also appears to be the predominant mechanism of target gene expression regulation directed by the miR408 sRNA. Moreover, the transcript abundance trends constructed for the *LAC3* target gene (Figure 5F), and the targeting miRNA, miR408 (Figure 4G), largely mirrored each other across the Col-0/NaCl, *drb1*/NaCl, and *drb2*/NaCl samples. In response to the significant increase in miR396 abundance (up 2.8-fold) in the Col-0/NaCl sample (Figure 4E), *GRF7* target gene expression was significantly reduced by 2.8-fold (Figure 5D) to show that in wild-type *Arabidopsis* seedlings, *GRF7* transcript abundance is controlled by the canonical, DRB1-dependent, and miR396-directed target transcript cleavage mode of RNA silencing. However, as stated above, the profiling of miR396 abundance in salt-stressed *drb1* and *drb2* seedlings suggested that the function of both DRB1 and DRB2 appears to be required for the complete control of miR396 production (Figure 4E). It is therefore of interest that the *GRF7* target gene expression analyses presented in Figure 5D additionally suggest that both DRB family members also play a role in determining the ability of miR396 to direct an appropriate level of gene expression regulation over its *GRF7* target gene. More specifically, although miR396 abundance remained largely unchanged in *drb1*/NaCl and *drb2*/NaCl seedlings, *GRF7* expression was significantly reduced in both mutant backgrounds following the application of salt stress—a target gene expression trend which indicated that the miR396-directed regulation of *GRF7* expression is defective in the absence of the activity of either DRB1 or DRB2. An opposing transcript abundance trend was also observed for miR399 and its *PHO2* target gene in salt-stressed Col-0 seedlings. Specifically, elevated miR399 abundance (Figure 4F), and decreased *PHO2* target gene expression was demonstrated by RT-qPCR in the Col-0/NaCl sample (Figure 5E). This miRNA target transcript abundance trend indicated that under salt stress conditions, miR399 regulates *PHO2* expression via the canonical DRB1-dependent transcript cleavage mode of RNA silencing. Furthermore, a DRB1-dependent, miRNA-directed target transcript cleavage mode of RNA silencing was confirmed as the predominant mode of the target gene expression regulation directed by the miR399 sRNA during salt stress, with RT-qPCR revealing *PHO2* gene expression to be elevated in the *drb1*/NaCl sample (Figure 5E) in the absence of change to the level of the targeting miRNA (Figure 4F). These miRNA accumulation and target gene expression trends show that the miR399-directed regulatory control of *PHO2* expression is rendered defective in the absence of DRB1 function. The mildly reduced abundance of both miR858 and its *ERF7* target transcript in the *drb1*/NaCl sample, where only DRB2 is functional, and the elevated miR858 abundance and decreased *ERF7* expression in the *drb2*/NaCl sample, where only DRB1 is functional, strongly infers that a DRB1-dependent miR858-directed target transcript cleavage mode, and a DRB2-dependent miR858-directed translational repression mode of RNA silencing, are both required to fine tune the abundance of the *ERF7* transcript in 15-day-old *Arabidopsis* seedlings.

## 4. Materials and Methods

### 4.1. Arabidopsis Plant Lines and Salt Stress Treatment Regime

The *Arabidopsis drb1* and *drb2* mutant lines, which harbor Transfer-DNA (T-DNA) insertion mutations in the *DRB1* (*drb1-1*; SALK_064863) and *DRB2* (*drb2-1*; GABI_348A09) genes, used in this study have been described previously [15,16,17]. The *drb* mutant seeds and those of unmodified wild-type Col-0 plants were surface-sterilized in a sealed container at room temperature for 90 min (min) using chlorine gas. Following sterilization, Col-0, *drb1,* and *drb2* seeds were plated out onto standard growth medium (half-strength Murashige and Skoog (MS) salts) for *Arabidopsis* cultivation. The plates were sealed with gas-permeable tape and incubated in the dark for 48 h (h) at 4 °C to stratify the seeds. Post stratification, the sealed plates were transferred to a temperature-controlled growth cabinet (A1000 Growth Chamber, Conviron^®^, Melbourne, Australia) and cultivated for an 8-day period under a standard growth regime of 16 h light/8 h dark, and a 22 °C/18 °C day/night temperature. At day 8, equal numbers (n = 48; 4 × plates of 12 seedlings per plate) of Col-0, *drb,1* and *drb2* seedlings were transferred to either (1) a fresh plate of growth medium (control plants (Ns plants)) or (2) a fresh plate of growth medium supplemented with 150 mM NaCl (salt-stressed plants (NaCl plants)). Following seedling transfer, the Ns and NaCl representative seedlings of each *Arabidopsis* line were returned to the temperature-controlled growth cabinet for an additional 7-day period of cultivation under the growth regime outlined above. It is important to note here that the concentration of NaCl used to supplement the growth medium, and the duration of the salt stress treatment period applied in this study, were selected to provide a prolonged mild-to-moderate phenotypic response in the three *Arabidopsis* lines under assessment based on previously reported experimentation [13,50,53]. In addition, for determination of primary root length, Ns and NaCl plants were treated exactly as outlined above except that the plates which housed the Col-0, *drb1,* and *drb2* seedlings were orientated vertically for the 7-day growth period following seedling transfer at day 8.

### 4.2. Phenotypic and Physiological Assessments

The fresh weight (milligrams (mg)) of 15-day-old Col-0, *drb1,* and *drb2* whole seedlings which were cultivated on either standard growth medium (Ns plants) or NaCl stress medium (NaCl plants) was recorded to determine the influence of the presence of 150 mM NaCl in the growth environment on *Arabidopsis* development. In addition, the rosette area (millimeters squared (mm^2^)) and primary root length (millimeters (mm)) of 15-day-old non-stressed and NaCl-stressed Col-0, *drb1,* and *drb2* plants were determined via the assessment of photographic images using the ImageJ software (https://imagej.net/ij/).

Anthocyanin content was determined according to [83]. In brief, 100 mg of whole seedlings was ground into a fine powder under liquid nitrogen (LN_2_) and incubated in 1.0 milliliter (mL) of acidic methanol (contained 1.0% *v/v* HCl) for 2 h at 4 °C. The ground plant material was centrifuged at 15,000 × *g* for 5 min at room temperature. The absorbance (A) of the resulting supernatant was measured at 530 (A_530_) and 657 (A_657_) nanometers (nm) in a GENESYS 10S spectrophotometer (Thermo Fisher Scientific, Sydney, Australia), with acidic methanol used as the blanking solution. The anthocyanin content of the non-stressed and NaCl-stressed Col-0, *drb1,* and *drb2* samples was determined by use of the equation: anthocyanin (μg/g FW) = A_530_ − 0.25 × A_657_/sample weight (g).

The chlorophyll *a* and *b* content of control-grown and salt-stressed 15-day-old Col-0, *drb1* and *drb2* seedlings was calculated according to [84]. Briefly, 100 mg of whole seedlings was ground into a fine powder under LN_2_ and then incubated in 1.0 mL of 80% (*v*/*v*) acetone in the dark for 24 h at room temperature. Following the incubation period, any remaining plant material was pelleted out of the solution by centrifugation at 15,000 × *g* for 5 min at room temperature. The absorbance (A_646_ and A_663_) of the resulting supernatant was measured at 646 and 663 nm in a GENESYS 10S spectrophotometer (Thermo Fisher Scientific, Sydney, Australia), with 80% acetone used as the blanking solution. Next, the chlorophyll *a* and *b* content of the non-stressed and NaCl-stressed Col-0, *drb1,* and *drb2* samples was determined using the Lichtenthaler’s equations exactly as outlined in [84], and these initially determined values were subsequently converted to micrograms per gram of fresh weight (μg/g FW).

### 4.3. Total RNA Extraction and Molecular Assessments

For the reported molecular assessments, total RNA was extracted from four biological replicates with each biological replicate consisting of a pool of 12 individual plants, using TRIzol™ Reagent according to the manufacturer’s protocol (Thermo Fisher Scientific, Sydney, Australia). It is important to note here that the plants used for total RNA extraction differed to those used for the physiological analyses due to the destructive nature of all experiments performed. The quality of the extracted total RNA was assessed via standard electrophoretic separation of nucleic acids on an ethidium bromide-stained 1.2% (*w*/*v*) agarose gel. For each high-quality total RNA preparation, a NanoDrop spectrophotometer (NanoDrop^®^ ND-1000, Thermo Fisher Scientific, Sydney, Australia) was subsequently employed to determine total RNA concentration in micrograms per microliter (μg/μL). A global, high molecular weight complementary DNA (cDNA) library for gene expression quantification was constructed via the digestion of 5.0 μg of total RNA with 5.0 units (U) of DNase I according to the instructions of the manufacturer (New England Biolabs, Melbourne, Australia). The DNase I-treated total RNA was next purified using a RNeasy Mini kit (Qiagen, Melbourne, Australia) according to the manufacturer’s protocol, and 1.0 μg of this purified preparation was then used as the template to synthesize cDNA via the use of 1.0 U of the ProtoScript^®^ II Reverse Transcriptase and 2.5 millimolar (mM) of oligo dT_(18)_ according to the manufacturer’s instructions (New England Biolabs, Melbourne, Australia). In addition, miRNA-specific cDNAs were synthesized via the treatment of 500 nanograms (ng) of total RNA with 0.5 U of DNase I (New England Biolabs, Melbourne, Australia), and each DNase I-treated total RNA sample was directly used as a template for miRNA-specific cDNA synthesis using miRNA-specific stem-loop DNA oligonucleotides (Table S2) and 1.0 U of ProtoScript^®^ II Reverse Transcriptase (New England Biolabs, Melbourne, Australia). The cycling conditions of (1) 1 cycle of 16 °C for 30 min, (2) 60 cycles of 30 °C for 30 s (s), 42 °C for 30 s, and 50 °C for 2 s, and (3) 1 cycle of 85 °C for 5 min were used for miRNA-specific cDNA synthesis. All generated single-stranded cDNAs were subsequently diluted to a working concentration of 50 ng/μL in RNase-free water prior to their use as a template for the quantification of the abundance of either a specific miRNA or a selected target gene of each quantified miRNA. All RT-qPCR assessments of transcript abundance used the same cycling conditions of (1) 1 cycle of 95 °C for 10 min and (2) 45 cycles of 95 °C for 10 s and 60 °C for 15 s. The GoTaq^®^ qPCR Master Mix (Promega, Sydney, Australia) was used as the fluorescent reagent for all performed RT-qPCR experiments, and miRNA abundance or gene transcript expression was quantified using the 2^−∆∆CT^ method with the small nucleolar RNA, snoR101, and *UBIQUITIN10* (*UBI10*; *AT4G05320*) used as the respective internal controls to normalize the relative abundance of each assessed transcript. For all RT-qPCR experiments reported here, four biological replicates were used per sample, and three technical replicates were performed per biological replicate. The sequence of each DNA oligonucleotide used in this study, either for the synthesis of a miRNA-specific cDNA or to quantify gene transcript abundance via RT-qPCR, is provided in Supplemental Table S2.

### 4.4. Statistical Analysis

Analytical data from this study were obtained from four biological replicates of the control and salt-stressed Col-0, *drb1*, and *drb2* samples, and each biological replicate consisted of a pool of 12 plants. Statistical analysis was performed using a standard two-tailed *t*-test. The presence of an asterisk (*) above a column of the graphs presented in Figure 1, Figure 2, Figure 4, and Figure 5, represents a statistically significant difference between the salt-stressed sample and its control-grown counterpart with *p*-values: * < 0.05, ** < 0.005, and *** < 0.001.

## 5. Conclusions

In this study, we attempted to provide experimental data which could demonstrate the likely reason that a DRB2-dependent miRNA pathway had evolved from the central and developmentally crucial DRB1-dependent miRNA pathway, was to offer *Arabidopsis* an alternate and specialized miRNA-mediated molecular response which could drive the phenotypic and physiological modifications required to adapt to abiotic stress, specifically salt stress. However, although *drb2* was determined to be the most sensitive to the imposed stress, each of the individual phenotypic and physiological parameters analyzed in this study differed across the three assessed plant lines at 15 days of age. Furthermore, at the molecular level, considerable differences were observed between salt-stressed Col-0 seedlings, and *drb1*/NaCl and *drb2*/NaCl plants. Namely, the large-scale trend of the up-regulated accumulation of miRNAs with altered abundance in salt-stressed Col-0 seedlings was not observed in either the *drb1* or *drb2* mutant background following the 7-day salt stress treatment period. The general trend of the reduced accumulation of miRNAs with altered abundance in *drb1*/NaCl and *drb2*/NaCl did however definitively demonstrate that *Arabidopsis* is defective in its ability to mount an appropriate miRNA-mediated molecular response to salt stress in the absence of the functional activity of either DRB1 or DRB2. Here, we have also provided evidence which demonstrates that both DRB1 and DRB2 direct differing regulatory roles to fine tune the production of individual miRNA species in both control-grown and salt-stressed *Arabidopsis* seedlings. Due to the requirement of the functional activity of both DRB1 and DRB2 for miRNA production, a combination of the canonical target transcript cleavage mode (directed by DRB1-dependent miRNAs) and the non-canonical translational repression mode (directed by DRB2-dependent miRNAs) of RNA silencing operates in *Arabidopsis* whole seedlings to add an additional layer of regulatory complexity to ensure the tightly controlled expression of each miRNA target gene, which would, in turn, ensure that the *Arabidopsis* plant can mount an appropriate miRNA-mediated molecular-based response to salt stress.

## Data Availability

All data reported here are available from the authors upon request.

## Supplementary Materials

The following supporting information can be downloaded at https://www.mdpi.com/article/10.3390/plants14060924/s1, Figure S1: Profiling of the abundance trends of miR160, miR164, miR167, miR396, miR399, miR408, and miR858 in control-grown Col-0, *drb1* and *drb2* seedlings; Table S1: Determination of whole family member abundance for miRNAs belonging to the *MIR160*, *MIR164*, *MIR167*, *MIR396*, *MIR399*, *MIR408,* and *MIR858* gene families in control-grown and salt-stressed Col-0, *drb1,* and *drb2* seedlings; Table S2: Sequences of the DNA oligonucleotides used in this study.
