# Supplementary material for: DRB1 and DRB2 Are Required for an Appropriate miRNA-Mediated Molecular Response to Salt Stress in Arabidopsis thaliana
Source: Plants (Basel). 2025 Mar 15;14(6):924. doi: 10.3390/plants14060924 (PMC11944917; doi:10.3390/plants14060924)
Supplement: Supplementary file 1 [file plants-14-00924-s001.zip › plants-3241547-supplementary.pdf]

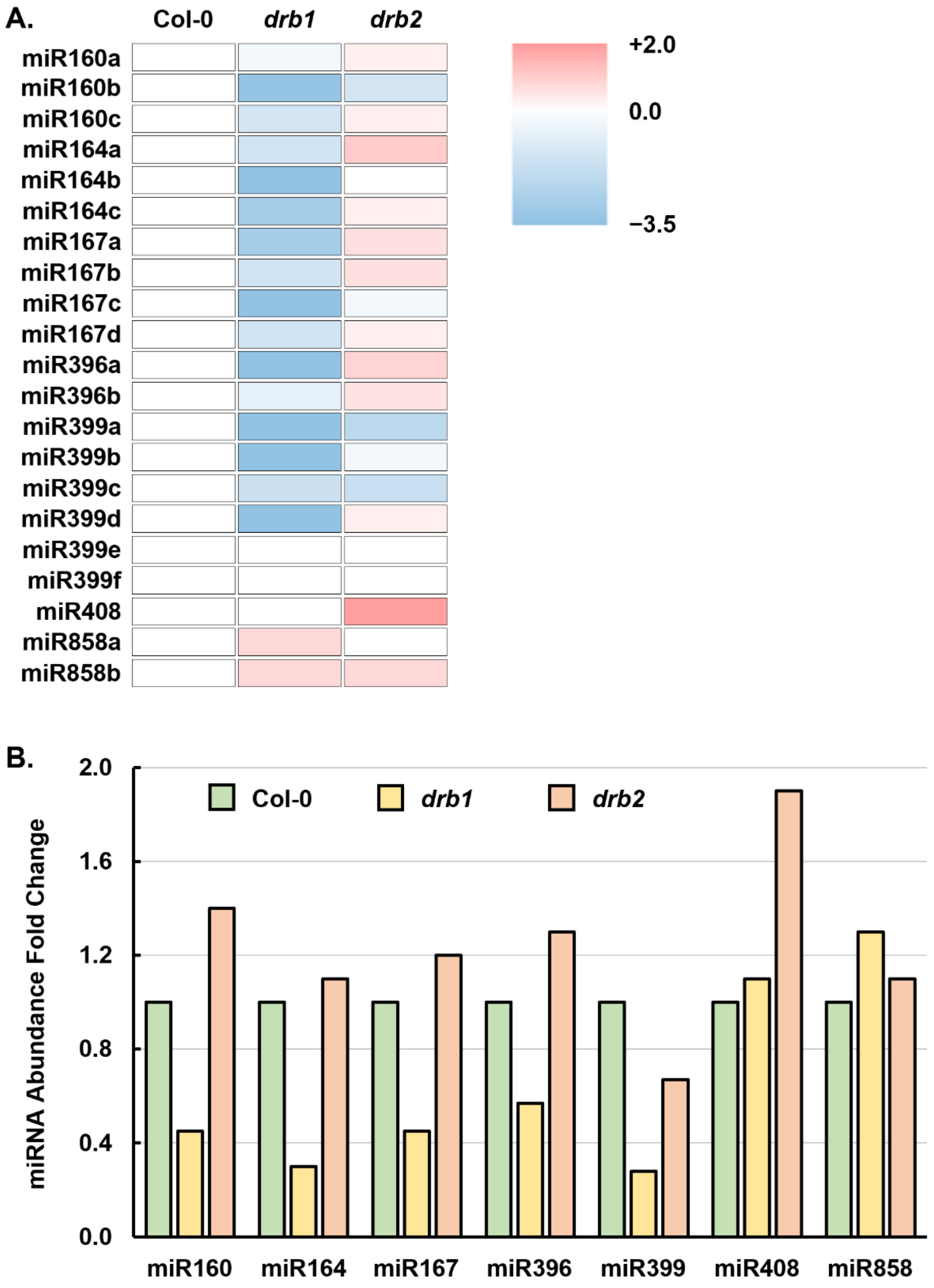

Figure S1. Profiling of the abundance trends of miR160, miR164, miR167, miR396, miR399, miR408 and miR858 in control grown Col-0, *drb1* and *drb2* seedlings. (**A**) The abundance trend of each individual member of the *MIR160*, *MIR164*, *MIR167*, *MIR396*, *MIR399*, *MIR408* and *MIR858* gene families in 15-day-old whole seedling samples of the Col-0, *drb1* and *drb2* plant lines cultivated under standard *Arabidopsis* growth conditions. The shading intensity of each tile in the heat map represents the degree of elevated (red colored shading) or reduced (blude colored shading) abundance (presented as a log2 fold change) of each *MIR* gene family member in the *drb1* or *drb2* mutant background compared to Col-0 seedlings of the same age. (**B**) Standard fold change assessment of the abundance of the 'summed total' of each individual family member detected by sRNA-Seq for miRNAs, miR160, miR164, miR167, miR396, miR399, miR408 and miR858, in the *drb1* (yellow columns) and *drb2* (orange columns) mutant backgrounds compared to 15-day-old wild-type *Arabidopsis* (Col-0) seedlings (green columns).

Table S1. Determination of whole family member abundance for miRNAs belonging to the *MIR160*, *MIR164*, *MIR167*, *MIR396*, *MIR399*, *MIR408* and *MIR858* gene families in control grown and salt stressed Col-0, *drb1* and *drb2* seedlings. The abundance of each family member was summed together to allow for subsequent comparison between the total sRNA-Seq read numbers for the *MIR160*, *MIR164*, *MIR167*, *MIR396*, *MIR399*, *MIR408* and *MIR858* gene families in the control grown samples Col-0/Ns, *drb1*/Ns and *drb2*/Ns samples and the salt stressed samples Col-0/NaCl, *drb1*/NaCl and *drb2*/NaCl. In Table S1, (+) symbol denotes elevated miRNA abundance in the salt stressed sample compared to the control, (-) symbol denotes reduced miRNA accumulation in the salt stressed sample compared to the control, and the (≈) symbol represents an equivalent degree of miRNA accumulation in both the control grown and salt stressed sample. In addition, Nd. shows that a *MIR* gene family member was not detected by the sRNA-Seq approach.

MIR160	***MIR* gene family member sRNA-Seq reads**						**Total Family Reads**	**Fold change (+/-)**
	**miR160a**	**miR160b**	**miR160c**					
Col-0/Ns	204	234	401				*839*	**+2.3**
Col-0/NaCl	351	257	1325				*1943*	
drb1/Ns	129	64	194				*387*	**-1.3**
drb1/NaCl	56	25	217				*298*	
drb2/Ns	285	301	579				*1165*	**≈1.0**
drb2/NaCl	232	242	681				*1155*	

MIR164	***MIR* gene family member sRNA-Seq reads**						**Total Family Reads**	**Fold change (+/-)**
	**miR164a**	**miR164b**	**miR164c**					
Col-0/Ns	94	677	175				*946*	**-1.4**
Col-0/NaCl	193	341	164				*698*	
drb1/Ns	48	164	65				*277*	**-2.3**
drb1/NaCl	33	60	30				*123*	
drb2/Ns	159	654	219				*1032*	**≈1.0**
drb2/NaCl	249	429	403				*1081*	

MIR167	***MIR* gene family member sRNA-Seq reads**						**Total Family Reads**	**Fold change (+/-)**
	**miR167a**	**miR167b**	**miR167c**	**miR167d**				
Col-0/Ns	3229	2896	1326	1890			*9341*	**+1.7**
Col-0/NaCl	5473	4959	1628	3681			*15741*	
drb1/Ns	1552	1510	366	849			*4277*	**-1.2**
drb1/NaCl	1341	1261	184	878			*3664*	
drb2/Ns	4108	3791	1076	2136			*11111*	**+1.3**
drb2/NaCl	5160	4739	1154	2865			*13918*	

MIR396	***MIR* gene family member sRNA-Seq reads**						**Total Family Reads**	**Fold change (+/-)**
	**miR396a**	**miR396b**						
Col-0/Ns	4316	7784					*12100*	**+1.7**
Col-0/NaCl	8549	12346					*20895*	
drb1/Ns	1078	5840					*6918*	**≈1.0**
drb1/NaCl	1127	6067					*7194*	
drb2/Ns	6480	9630					*16110*	**+1.2**
drb2/NaCl	8694	10322					*19016*	

MIR399	*MIR* gene family member sRNA-Seq reads						Total Family Reads	Fold change (+/-)
	**miR399a**	**miR399b**	**miR399c**	**miR399d**	**miR399e**	**miR399f**		
Col-0/Ns	386	322	341	8	4	Nd.	*1061*	**+4.0**
Col-0/NaCl	2997	550	579	58	10	Nd.	*4194*	
drb1/Ns	104	96	98	1	0	Nd.	*299*	**+1.1**
drb1/NaCl	268	35	30	0	0	Nd.	*333*	
drb2/Ns	210	237	240	12	1	Nd.	*700*	**+2.5**
drb2/NaCl	1109	290	317	48	6	Nd.	*1770*	
MIR408	***MIR*** **gene family member sRNA-Seq reads**						**Total Family Reads**	**Fold change (+/-)**
	miR408							
Col-0/Ns	2242						*2242*	**+5.5**
Col-0/NaCl	12273						*12273*	
drb1/Ns	2436						*2436*	**+1.7**
drb1/NaCl	4180						*4180*	
drb2/Ns	4322						*4322*	**+2.1**
drb2/NaCl	8881						*8881*	
MIR858	***MIR*** **gene family member sRNA-Seq reads**						**Total Family Reads**	**Fold change (+/-)**
	miR858a	miR858b						
Col-0/Ns	79	4					*83*	**+1.0**
Col-0/NaCl	146	13					*159*	
drb1/Ns	101	7					*108*	**-1.3**
drb1/NaCl	73	12					*85*	
drb2/Ns	82	7					*89*	**+2.3**
drb2/NaCl	194	13					*207*	

Table S2. Sequences of the DNA oligonucleotides used in this study.

Target analyzed	Oligonucleotide name	Oligonucleotide sequence (5′ to 3′)
miRNA-specific cDNA synthesis and RT-qPCR analysis		
miR160	p-mir160-RTF	CGCCTGACAGAAGAGAGTGAGCAC
	p-mir160-RTR	GTCGTATCCAGTGCAGGGTCCGAGGTATTCGCACTGGATACGACGTGCTC
miR164	p-mir164-RTF	GGCTGGAGAAGCAGGGCACGTGCA
	p-mir164-RTR	GTCGTATCCAGTGCAGGGTCCGAGGTATTCGCACTGGATACGACTGCACG
miR167	p-mir167-RTF	CGCTGAAGCTGCCAGCATGATCTA
	p-mir167-RTR	GTCGTATCCAGTGCAGGGTCCGAGGTATTCGCACTGGATACGACCGGCAA
miR396	p-mir396-RTF	GCGCGTTCCACAGCTTTCTTGAAC
	p-mir396-RTR	GTCGTATCCAGTGCAGGGTCCGAGGTATTCGCACTGGATACGACAAGTTC
miR399	p-mir399-RTF	GCATGCCAAAGGAGATTTGCCCTG
	p-mir399-RTR	GTCGTATCCAGTGCAGGGTCCGAGGTATTCGCACTGGATACGACCAGGGC
miR408	p-mir408-RTF	ACGACAGGGAACAAGCAGAGCATG
	p-mir408-RTR	GTCGTATCCAGTGCAGGGTCCGAGGTATTCGCACTGGATACGACCATGCT
miR858	p-mir858-RTF	GGCGTTTCGTTGTCTGTTCGACCTT
	p-mir858-RTR	GTCGTATCCAGTGCAGGGTCCGAGGTATTCGCACTGGATACGACAAGGTC
Generic Stem-loop	p-generic-STL	CCAGTGCAGGGTCCGAGGTA
snoR101	p-snor101-RTF	CTTCACAGGTAAGTTCGCTTG
	p-snor101-RTR	AGCATCAGCAGACCAGTAGTT
High molecular weight cDNA synthesis and RT-qPCR analysis		
Ath-DCL1	p-dcl1-RTF	AATGGGCATCAGCCGTTTACGAGA
	p-dcl1-RTR	AAATCTCTTTGCATGAGCCGGTCC
Ath-DRB1	p-drb1-RTF	ATGACCTCCACTGATGTTTCC
	p-drb1-RTR	TGCTAATTCCCGGAGAGC
Ath-DRB2	p-drb2-RTF	ATGTATAAGAACCAGCTACAAGAGTTG
	p-drb2-RTR	CAGCAGCAGAGTGTTCAGC
Ath-P5CS1	p-p5cs1-RTF	GTTTTTGAATCCCGACCTGA
	p-p5cs1-RTR	TTACCCCCAACAGTCTCTGG
Ath-UBI10	p-ubi10-RTF	GGCCTTGTATAATCCCTGATGAATAAG
	p-ubi10-RTR	AAAGAGATAACAGGAACGGAAACATA
Ath-ARF8	p-arf8-RTF	GGTTGGGCGTTCATTAGACA
	p-arf8-RTR	ATGTACCAAACGTTATTCACA
Ath-ARF17	p-arf17-RTF	CGAGTCAAGATGGCTATGGA
	p-arf17-RTR	CATCCCATGTGATCTGAAGC
Ath-CUC1	p-cuc1-RTF	CTTCTTCTTCTGCCGTCACC
	p-cuc1-RTR	GAGCGGGAAGGAATGTATGA
Ath-GRF7	p-grf7-RTF	CATCCCCCACCGTTAGATCG
	p-grf7-RTR	TGCTTCCATGCTTCCGACAT
Ath-PHO2	p-pho2-RTF	ACCGTTTCTCATCAAGGCGT
	p-pho2-RTR	GTGCCCGTCCACCATAAGAA
Ath-LAC3	p-lac3-RTF	CCGTTCGACAACACAACCAC
	p-lac3-RTR	GACTGGGAAAACAGGAGCGA
Ath-ERF7	p-erf7-RTF	CCGTGGCATAGAAGCAAAGT
	p-erf7-RTR	CGGTGAGTGGTTTTGTTGTG
poly-A tail	p-oligo-dT23	TTTTTTTTTTTTTTTTTT
